# Multi-Method Screening of Commercial Antioxidant Supplements: Antioxidant Activity and Selected Antioxidant Compound Profiles

**DOI:** 10.3390/foods15081303

**Published:** 2026-04-09

**Authors:** Iwona Mirończuk-Chodakowska, Karolina Kujawowicz, Monika Cyuńczyk, Monika Sejbuk, Anna Maria Witkowska

**Affiliations:** Department of Food Biotechnology, Medical University of Bialystok, Szpitalna 37, 15-295 Bialystok, Poland; karolina.kujawowicz@umb.edu.pl (K.K.); monika.cyunczyk@umb.edu.pl (M.C.); monika.sejbuk@sd.umb.edu.pl (M.S.); anna.witkowska@umb.edu.pl (A.M.W.)

**Keywords:** supplements, antioxidant, polyphenol

## Abstract

Daily diets may not always provide adequate amounts of antioxidants for some individuals, prompting a growing interest in dietary antioxidant supplements. This study focuses on a diverse range of supplements marketed as “antioxidant supplements” in the Polish market, encompassing both single-ingredient and multi-component formulations. The objective of this study was to assess the antioxidant activity and polyphenolic profiles of these supplements to provide comparative insights. A total of 96 supplements from various manufacturers were analyzed. Polyphenol content was determined using the Folin–Ciocalteu method, while antioxidant activity was evaluated using three approaches: FRAP, DPPH, and electrochemical assays, together with HPLC-based profiling of selected antioxidant compounds. Total polyphenol content ranged from 0.146 to 177.499 mg GAE/g. Antioxidant activity showed broad dispersion across products, with FRAP values from 0.027 to 64.734 mmol/g, DPPH from 0.013 to 22.499 mmol Trolox/g, and electrochemical response from 51.162 to 374 µC. FRAP and DPPH were strongly correlated with each other (Spearman ρ ≈ 0.68) and with total polyphenols (ρ ≈ 0.89 and 0.84, respectively). Electrochemical response showed moderate correlation with total polyphenols (ρ ≈ 0.63). Moderate positive correlations were observed between assay responses and rutin, quercetin, resveratrol, ferulic acid, and chlorogenic acid. Compound profiles differed between supplement source groups and were highly heterogeneous in multi-component formulations. The results indicate that antioxidant activity is associated with compound composition and distribution rather than total polyphenol content alone and support the use of multiple complementary assays for supplement evaluation.

## 1. Introduction

Antioxidants play a crucial role in the prevention and treatment of chronic and neurodegenerative diseases due to their wide range of biological properties in the human body [1,2,3]. Their primary and most important function is the ability to neutralize reactive oxygen species (ROS) and free radicals. However, daily diets, which vary widely depending on individual, cultural, and lifestyle factors, may not always provide adequate levels of antioxidants for certain individuals. Consequently, some populations may need supplementation to support their body’s antioxidant defenses.

Over the past two decades, the use of dietary supplements has increased significantly. The supplement market has not only grown in sales but also expanded substantially in the variety of products available. According to recent reports, the market for dietary supplements continues to experience steady growth [4]. According to recent reports, the global market is expected to be worth $300 billion by 2028 [5].

In Poland, the dietary supplement market is experiencing significant growth, with online sales accounting for 20% of total revenue in 2024 [6]. The market offers a diverse range of supplements, many of which are marketed as antioxidant supplements.

These products can be categorized based on their chemical structure into several groups, including vitamins, carotenoids, minerals, and polyphenols, which may be either synthetic or naturally derived. Antioxidant supplements are commonly available in the form of powders and extracts. They can be classified as either single-ingredient (containing one active substance) or multi-ingredient formulations (combining multiple active substances along with vitamins and minerals).

The increasing consumer awareness of the health benefits of antioxidants has contributed to the expansion of the antioxidant supplement market. In Poland, these supplements are widely available in pharmacies, herbal shops, online stores, and, occasionally, grocery stores. To meet consumer demand for optimal antioxidant products, manufacturers are actively developing innovative formulations. Numerous studies have examined the antioxidant properties of food products [7,8]. However, despite the growing market for dietary supplements, knowledge regarding their antioxidant activity remains limited. Many of these supplements are multicomponent, and the combined effects of their ingredients are still not well understood. Unlike pharmaceuticals, dietary supplements—classified as a specific category of food—are not subjected to the rigorous and time-consuming testing required for drugs. Nevertheless, they must comply with safety standards, quality controls, and labeling regulations to ensure consumer protection [9,10,11]. Despite these regulations, low-quality, contaminated, or adulterated formulations occasionally reach the market [12,13].

The antioxidant activity of these formulations remains inadequately studied [14,15]. Product development is typically based on the existing literature and ingredient availability rather than direct evaluation of the final product’s antioxidant efficacy. This issue is particularly relevant for multicomponent formulations, where ingredient interactions can influence overall antioxidant potential. Various methods are available to assess antioxidant activity, with the spectrophotometric FRAP (Ferric-reducing Antioxidant Power) and DPPH (2,2-diphenyl-1-picrylhydrazyl) methods being the most widely used. These assays represent complementary mechanisms, reflecting reducing capacity (FRAP) and radical scavenging activity (DPPH), and are commonly applied in comparative screening studies [8]. For polyphenol quantification, the Folin–Ciocalteu method is commonly applied. Many studies on antioxidant activity and polyphenols, as well as related databases [7,16], rely on these well-established techniques, allowing for standardized comparisons across different products. A growing area of interest is electrochemical analysis, which directly measures antioxidant activity using electrodes. This technique aligns with sustainability principles by eliminating the need for reagents and enabling real-time measurements. Additionally, it provides an efficient alternative to traditional methods, making it particularly valuable for screening antioxidant properties in research settings.

However, studies combining multiple complementary analytical approaches with compound-level profiling across a broad range of commercial antioxidant supplements remain limited. In particular, there is a lack of comparative studies that relate antioxidant activity to the composition of selected bioactive compounds in complex, multi-component formulations.

Therefore, the aim of this study was to perform a multi-method screening of dietary supplements marketed as antioxidant products on the Polish market. The analysis covered both single-ingredient and multi-component formulations and combined global antioxidant assays (FRAP, DPPH), electrochemical methods, determination of total polyphenols, and HPLC-based profiling of selected marker compounds. The study provides a comparative overview of antioxidant properties across a diverse set of commercially available products.

## 2. Materials and Methods

### 2.1. Sample Collection

The study included 96 commercially available dietary supplements marketed as antioxidant products on the Polish market. The selection comprised both single-ingredient and multi-component formulations obtained from various manufacturers.

Products were identified through a structured search using the keyword “antioxidant supplements” across widely used Polish online retailers, ensuring broad market coverage. A total of 21 manufacturers were included.

The supplements were categorized according to the dominant declared raw material or product concept into nine groups (e.g., fruit-based, coffee- and tea-derived, mushroom-based, root-based, bark-based, leaf- and herb-based, algae-based, and multi-ingredient formulations). Products were assigned to a given category based on the primary declared component or product identity, even if additional ingredients were present.

**Figure A1:**
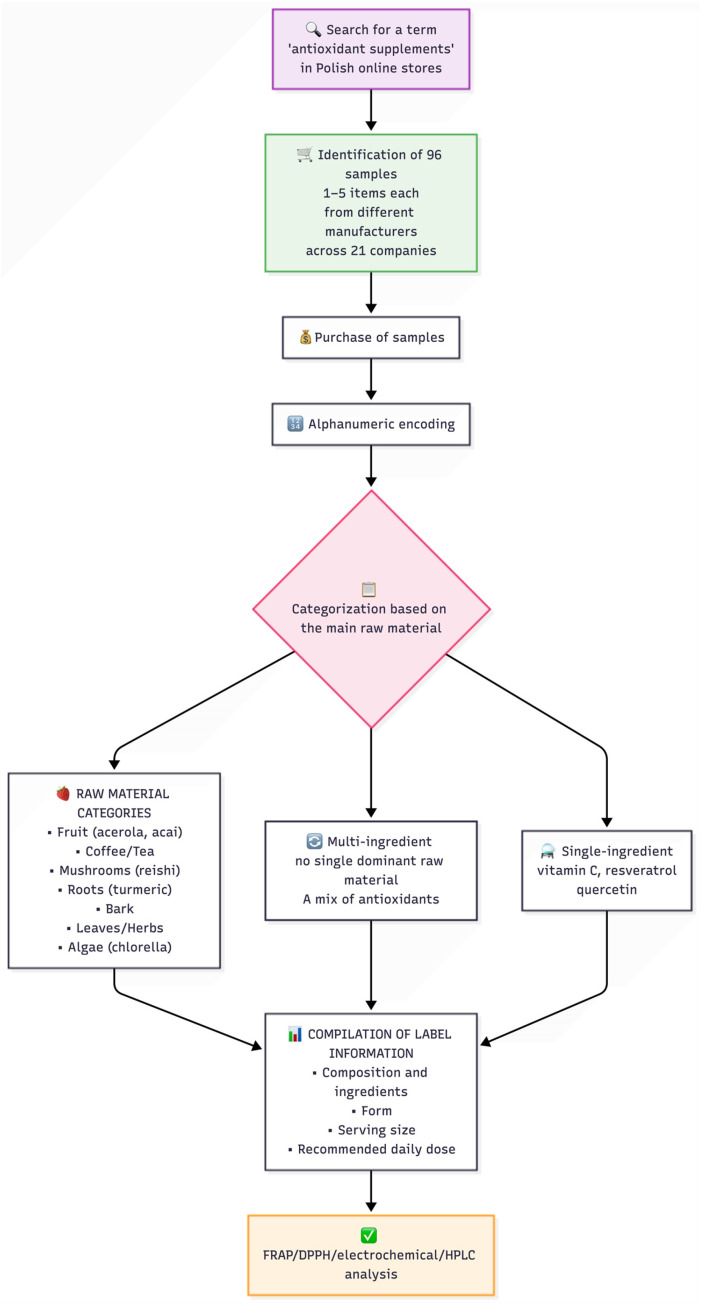
Sample selection flowchart.

From each category, 1 to 5 representative products were selected. Each supplement was assigned a unique alphanumeric code to ensure traceability. A detailed flowchart of the selection procedure is provided in Appendix A (Figure A1).

The composition of bioactive substances (excluding excipients such as anti-caking agents, colorants, and fillers) is presented in Appendix A (Table A1), while an alphabetical list of manufacturers is given in Table A2.

To ensure a neutral presentation of the results and avoid product-specific bias and potential commercial interpretation, individual product names were not disclosed.

### 2.2. Sample Preparation

For each supplement, 500 mg was weighed, and 10 cm^3^ of a methanol–water solution (9:1) was added. The samples were mixed on a shaker for 180 min at room temperature and then refrigerated for 20 h due to laboratory scheduling. This standardized procedure ensures consistent sample treatment for accurate analysis. Following refrigeration, the samples were centrifuged for 10 min and filtered through filter paper. The resulting extracts were then used for subsequent analyses. Samples were prepared and measured in duplicate.

### 2.3. Measurement of Total Polyphenol Content

The total polyphenol content of the supplements was determined using the Folin–Ciocalteu method, as described by Singleton and Rossi [17]. This method is based on the oxidation of phenolic compounds by the Folin–Ciocalteu reagent, which contains phosphotungstic and phosphomolybdic acids. The reagent undergoes partial reduction, forming a molybdenum–tungsten blue complex, which is measured spectrophotometrically at 765 nm and expressed as gallic acid equivalents (GAE).

In this procedure, 0.4 mL of the extracted sample was mixed with 2 mL of Folin–Ciocalteu reagent, previously diluted 1:5 in distilled water. Afterward, 1.6 mL of 7.5% (*w*/*v*) sodium carbonate was added. The absorbance was measured at 765 nm using a Shimadzu UV-1800 spectrophotometer (Shimadzu, Kyoto, Japan) after 30 min of reaction time. The results were calculated based on the solid mass of the analyzed samples and expressed as mg GAE/g. All experiments were performed in duplicate to ensure accuracy and reproducibility.

### 2.4. FRAP Assay

The FRAP (Ferric Reducing Antioxidant Power) assay was performed according to [18]. This method is based on the reduction of the Fe^3+^–2,4,6-tripyridyl-s-triazine (TPTZ) complex to its TPTZ-Fe^2+^ form.

A freshly prepared FRAP reagent (3.0 mL) was heated to 37 °C, and a reagent blank was measured at 593 nm (A_1_) using a Shimadzu UV-1800 spectrophotometer (Shimadzu, Kyoto, Japan). Subsequently, 100 μL of the sample and 300 μL of distilled water were added to the FRAP reagent. After incubating the mixture at 37 °C for 4 min, the absorbance (A_2_) was measured. The antioxidant potential of the sample was determined by referencing a standard curve constructed using FeSO_4_·7H_2_O at concentrations ranging from 100 to 1000 μmol/L. The reported data represent the means of two measurements.

### 2.5. DPPH Method

The determination of antioxidant properties using the DPPH method was carried out according to the procedure by Brand-Williams [19] and modified by Thaipong [20]. It involves the reduction of the stable azo radical DPPH by antioxidants contained in the sample. The alcoholic solution of the DPPH radical is purple in color with a maximum absorbance at 515 nm. DPPH radical solutions at a concentration of 0.10 mmol/L (prepared from a 0.60 mmol/L solution in methanol) were obtained immediately before analysis. Their absorbance was 1.12 ± 0.05. During the analysis, 50 µL of the extract was mixed with 1450 µL of 0.10 mmol/L DPPH solution and kept in an incubator at 27 °C for 60 min, after which the absorbance of the discolored solutions was measured. Quantification was performed using the calibration curve method. The curves were constructed based on the results of absorbance determinations of 6 concentrations (ranging from 0.0781 mmol/L to 2.5 mmol/L) of methanolic solutions of Trolox. The reported data represent the means of two measurements.

### 2.6. Electrochemical Method

This method measures a sample’s ability to counteract oxidation using an eBQC apparatus (Bioquochem, Asturias, Spain). The sample is placed on an electrode, and an electrical potential is applied. Antioxidants in the sample donate or accept electrons, generating a current that is recorded. The magnitude of this current correlates with the quantity and activity of antioxidants present in the sample. The results are expressed in microcoulombs (µC).

### 2.7. HPLC Method

All tested samples were solid dietary supplements. Each sample was finely grounded in a ceramic mortar, and approximately 0.5 g of each sample was accurately weighed and extracted with 10 mL of 90% methanol. Extractions were carried out on a rotary platform shaker for 180 min at room temperature. The extracts were centrifuged, and the resulting supernatants were filtered through 0.45 µm PTFE syringe filters prior to HPLC analysis. Blank samples consisting of extraction solvent were processed identically to test samples and showed no detectable peaks at analyte retention times.

The HPLC analyses were conducted on a Shimadzu Prominence system equipped with an LC-20AD binary pump, SPD-M20A diode array detector (DAD), and a Rheodyne manual injection valve fitted with a 20 µL loop. Data acquisition and analysis were performed using LabSolutions software, version 1.22 SP1. Separations were performed using a Synergi Hydro-RP C18 reversed-phase column (250 mm × 4.6 mm, 5 µm particle size; Phenomenex, Torrance, CA, USA).

Two chromatographic methods were applied. In Method 1, used for the determination of ascorbic acid, gallic acid, ferulic acid, rutin, quercetin, lipoic acid, resveratrol, and curcumin, the mobile phase consisted of water with 1% formic acid (solvent A) and acetonitrile (solvent B). Elution was performed using a linear gradient from 95% A to 5% A over 40 min, followed by column re-equilibration. The total run time was 55 min. The flow rate was 0.8 mL/min, the injection volume was 20 µL, and the column was maintained at 30 °C.

In Method 2, applied to chlorogenic acid and catechin, the mobile phases were water with 0.1% formic acid (A) and acetonitrile (B). A gradient elution was applied, decreasing from 95% A to 82% in 30 min, followed by column re-equilibration. The total run time was 40 min. The flow rate and injection volume were the same as in Method 1, and the column temperature was set at 35 °C.

Calibration curves were constructed for each analyte using weighted (1/x) linear regression of peak area versus concentration, to account for heteroscedasticity across the calibration range. Quantification was performed using the DAD at compound-specific wavelengths. Limits of detection (LOD) and limits of quantification (LOQ) were calculated as 3.3σ/s and 10σ/s, respectively, where σ represents the residual standard deviation of the regression and s is the slope of the calibration curve. Detection wavelengths, retention times, and calculated LOD and LOQ values are summarized in Appendix B Table A3 and Table A4.

Calibration was based on external standards using seven concentration levels ranging from 0.5 to 100 µg/mL. Measured values were expressed in µg/mL of extract and recalculated per mg of supplement. For samples requiring dilution prior to injection, the results were corrected using the appropriate dilution factor. Values below the detection limit were reported as ND (not detected), and values between LOD and LOQ were reported as <LOQ. LOD was treated strictly as a qualitative threshold, and analytes below this level were not quantified. For statistical purposes, ND values were treated as zero and <LOQ values were substituted with LOQ/2. Numerical concentrations were reported only for results above the quantification limit.

Repeatability of the chromatographic system, evaluated based on six replicate injections of standard solutions, showed %RSD values below 3% for representative compounds, indicating acceptable analytical repeatability under the applied conditions.

The chromatographic analysis was intended for comparative profiling rather than full quantitative method validation.

### 2.8. Visualization

**Figure A2:**
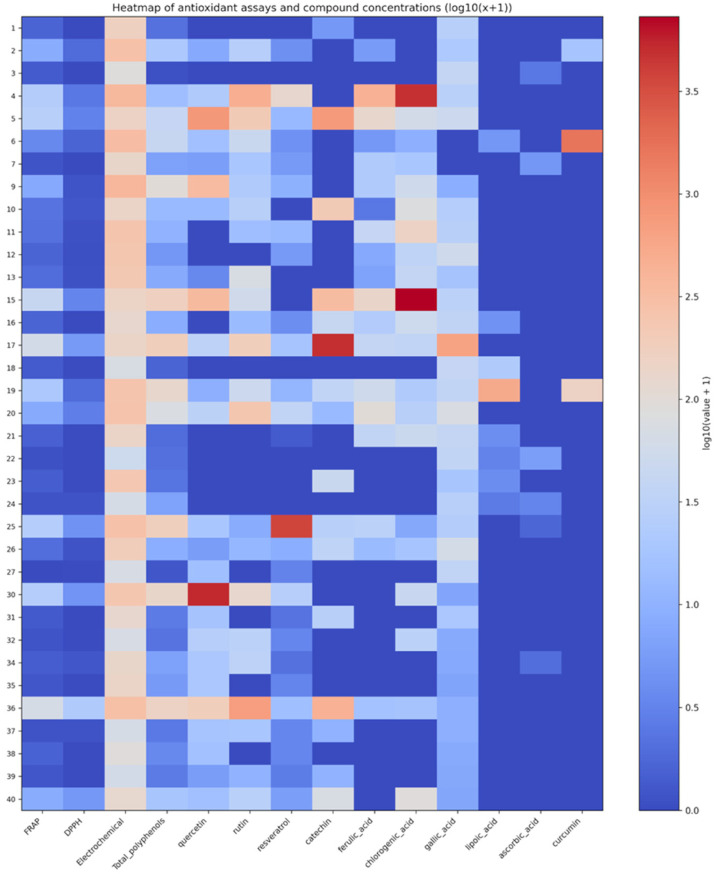
Summary heatmap of antioxidant assay results and quantified compound concentrations across samples. Values were log10(x + 1)-transformed prior to visualization to reduce dynamic range and preserve zero values. Rows represent samples and columns represent antioxidant assays (FRAP, DPPH, Electrochemical), total polyphenols, and individual compounds. Colors indicate relative magnitude of each parameter. Sample IDs are sequential laboratory codes; not all codes are represented in this dataset.

Results of antioxidant assays and compound concentrations were visualized using boxplots, scatter plots, and heatmaps. Two types of heatmaps were generated for different analytical purposes. Heatmaps presenting compound and assay profiles across samples or supplement groups were based on measured values. For these visualizations, variables with skewed distributions were log10(x + 1) transformed to reduce dynamic range and retain zero values (Appendix C Figure A2). The transformed data were then column-wise standardized using z-score transformation to enable comparison across variables measured in different units and scales. A continuous color scale was applied to represent relative differences.

**Figure A3:**
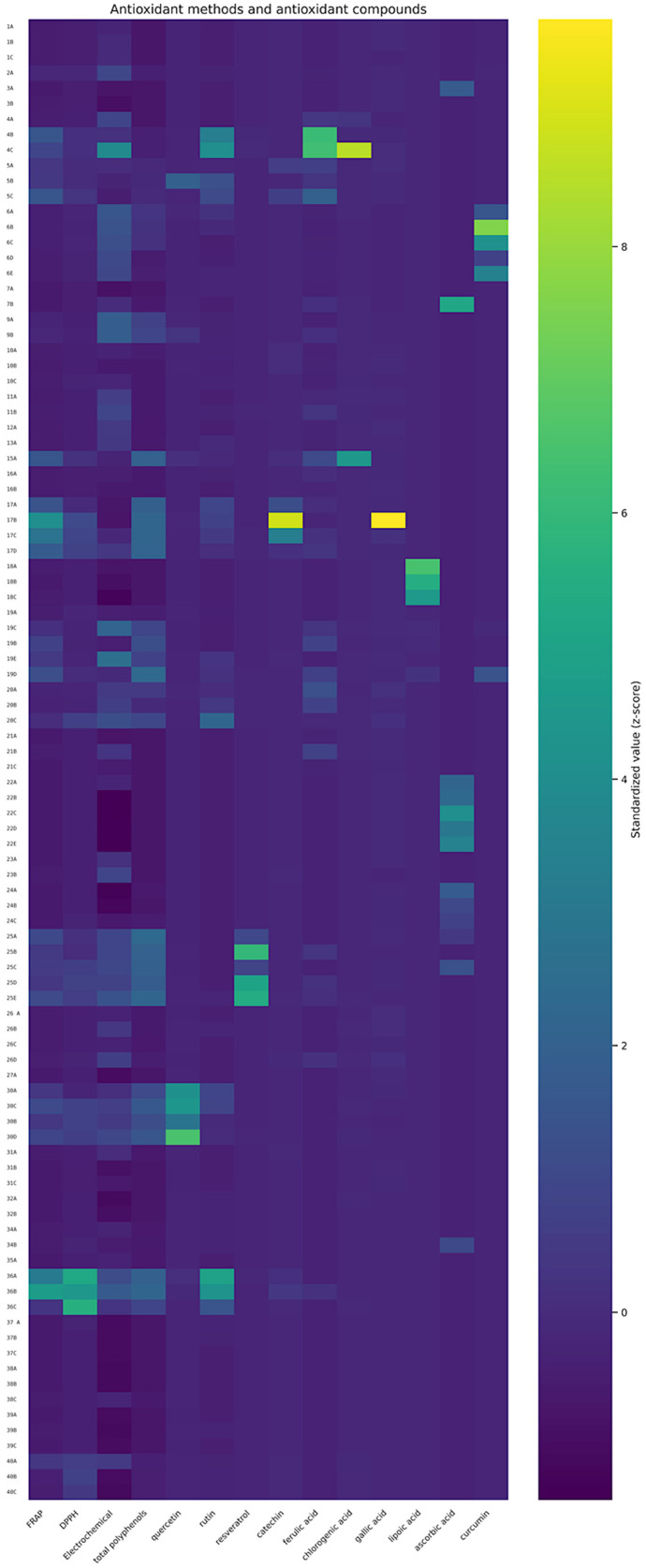
Heatmap of antioxidant method results and quantified compound concentrations across samples.

Correlation heatmaps were constructed from Spearman correlation coefficients calculated between antioxidant assays, total polyphenols, and selected compounds. These heatmaps were generated directly from correlation values and were not log-transformed or z-score standardized (Appendix C Figure A3).

### 2.9. Statistical Analysis

The normality of distribution for quantitative variables was assessed using the Shapiro–Wilk test. Since the data did not follow a normal distribution, Spearman’s rank correlation test was used to analyze relationships between quantitative variables. Statistical significance was set at *p* < 0.05. All statistical analyses were performed using Statistica 13.0 (StatSoft, Inc., Tulsa, OK, USA) and Microsoft Excel.

## 3. Results

The study analyzed 96 dietary supplement samples, the majority of which were in capsule (85) and tablet (7) form, with 4 supplements available as spoon-measured powders. Among the capsule and tablet formulations, 42 contained plant extracts, while the remaining samples consisted of powdered plant materials. The study also included multi-ingredient formulations containing powdered fruits, herbs, plant extracts, antioxidant vitamins, B vitamins, micronutrients, and amino acids.

Table 1, Table 2, Table 3, Table 4, Table 5, Table 6, Table 7, Table 8 and Table 9 present the mean, median, minimum, and maximum values for polyphenol content and antioxidant activity, measured using FRAP, DPPH and electrochemical methods, across the following supplement groups: (1) fruit-based supplements, (2) supplements containing coffee or tea, (3) mushroom-based supplements, (4) root-based supplements, (5) bark-based supplements, (6) green plant-based supplements, (7) algae-based supplements, (8) single-ingredient supplements, and (9) multi-ingredient supplements. Polyphenol content is expressed in mg GAE/g, while antioxidant activity is reported in mmol/g (FRAP method); also, antioxidant activity is reported in mmol Trolox/g (DPPH method) and µC (electrochemical method).

In fruit-based supplements (Table 1), polyphenol content ranged from 0.146 mg/g in apple cider vinegar (3A, 3B) to 177.499 mg/g in grape seed extract (17A–17D). Apple polyphenol extract (15A) also exhibited a high average polyphenol content of 169.695 mg/g. Grape seed extract supplements (17A–17D) demonstrated high antioxidant activity, with average values of 60.842 mmol/g (FRAP), 4.419 mmol Trolox/g (DPPH) and 141.8 µC (electrochemical method).

**Figure 1:**
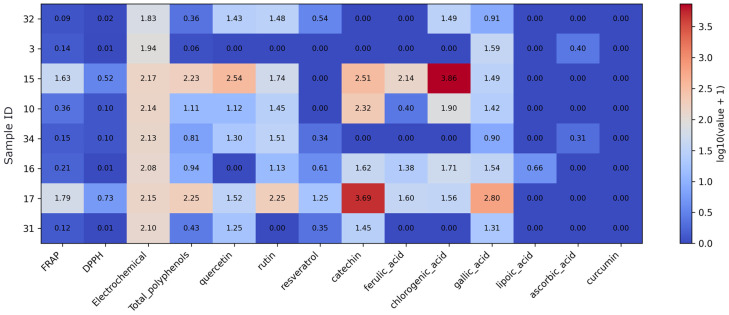
Heatmap of antioxidant assay results and quantified antioxidant compound concentrations in the fruit-based supplement subset. Note: Values were log10(x + 1)-transformed prior to visualization to reduce dynamic range and preserve zero values. As the data originate from different analytical methods and units, the transformation allows comparative visualization rather than direct quantitative comparison.

Within the fruit-based supplement subset, the heatmap (Figure 1) summarizes antioxidant activity and selected compound profiles and shows substantial variability between samples. Values were log-transformed (log10(value + 1)) to reduce data skewness and enable better visualization of differences across samples and variables. Samples 15 (apple polyphenol extract) and 17 (grape seed extract) exhibit the highest total polyphenol content and the strongest FRAP and DPPH values, together with elevated levels of catechin and chlorogenic acid. Sample 10 (black chokeberry extract) is distinguished by high catechin concentration with intermediate assay responses. In contrast, samples 3 (apple cider powder), 31 (lycopene from tomato extract), and 32 (açaí berry powder) display consistently low polyphenol and flavonoid levels and correspondingly low antioxidant assay values. Rutin and chlorogenic acid show the greatest variability across fruit-based products, whereas lipoic acid, ascorbic acid, and curcumin are largely absent in this group.

Among coffee- and tea-based supplements (Table 2), green tea leaf extract exhibited a higher average polyphenol content (150.991 mg/g) and strong antioxidant activity measured by FRAP method (64.734 mmol/g) and DPPH method (22.499 mmol Trolox/g). However, when antioxidant activity was assessed using the electrochemical method, the results differed. Green coffee extracts and powders demonstrated higher antioxidant activity, with a mean of 354.8 µC.

**Figure 2:**
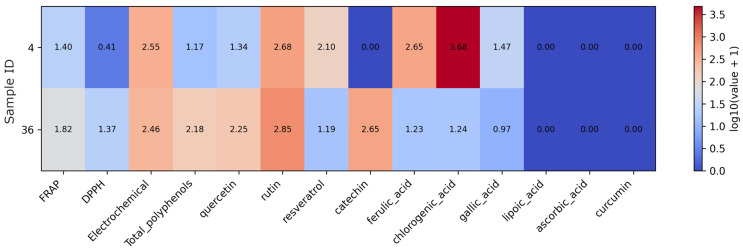
Heatmap of antioxidant assay results and quantified antioxidant compound concentrations in the coffee- or tea-based supplement subset. Note: Values were log10(x + 1)-transformed prior to visualization to reduce dynamic range and preserve zero values. As the data originate from different analytical methods and units, the transformation allows comparative visualization rather than direct quantitative comparison.

Log transformation (log10(value + 1)) was applied to reduce the influence of extreme values and facilitate visualization of data spanning different orders of magnitude in the subset of coffee- and tea-based supplements (Figure 2). The green tea supplement shows (sample 36) higher total polyphenols, FRAP, and DPPH values than the green coffee product (sample 4). It is also characterized by higher quercetin, rutin, and catechin levels. In contrast, the green coffee supplement displays markedly higher chlorogenic and ferulic acid concentrations together with a stronger electrochemical response. Lipoic acid, ascorbic acid, and curcumin are not detected in either product.

Among mushroom-based supplements (Table 3), reishi mushroom extracts and powders (38A–38C) exhibited the highest average polyphenol content and antioxidant activity, as measured by both the FRAP and electrochemical methods. The mean values were 2.534 mg GAE/g, 0.61 mmol/g (FRAP), and 89.796 µC (electrochemical method). In contrast, chaga mushrooms showed the lowest average polyphenol content (0.239 mg/g) and lowest antioxidant activity (0.027 mmol/g, FRAP method). The lowest antioxidant activity measured by the electrochemical method was observed in lion’s mane (37A–37C). Antioxidant activity measured by the DPPH method ranged from 0.043 mmol Trolox/g in reishi mushroom to 0.179 mmol Trolox/g in lion’s mane mushroom.

**Figure 3:**
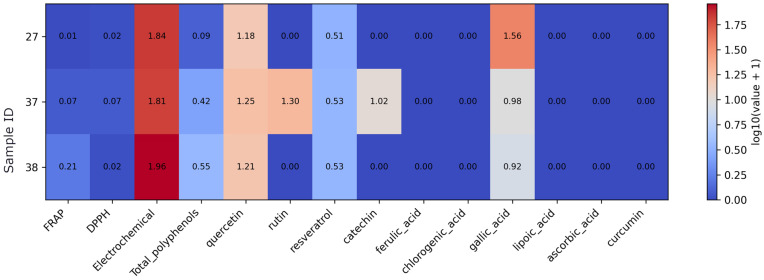
Heatmap of antioxidant assay results and quantified antioxidant compound concentrations in mushroom supplements. Note: Values were log10(x + 1)-transformed prior to visualization to reduce dynamic range and preserve zero values. As the data originate from different analytical methods and units, the transformation allows comparative visualization rather than direct quantitative comparison.

In mushroom-based supplements, antioxidant assay responses are generally low for FRAP and DPPH, while electrochemical signals are relatively higher and consistent across all three products (Figure 3). Total polyphenol levels remain low in this group. Quercetin and resveratrol are detected in all samples at comparable levels, whereas rutin and catechin appear only in a single product (sample 37, lions’ mane). Gallic acid is present in all three supplements and represents the dominant quantified compound in this subset, while ferulic acid, chlorogenic acid, lipoic acid, ascorbic acid, and curcumin are not detected.

Among root-based plant supplements (Table 4), the average polyphenol content ranged from 1.730 mg GAE/g in maca (39A–39C) to 173.865 mg GAE/g in resveratrol (25A–25E). Curcuma longa-based supplements (6A–6E) also exhibited high polyphenol content, with an average of 41.370 mg GAE/g. In terms of antioxidant activity, Curcuma longa-based supplements demonstrated the highest average value as measured by the electrochemical method (312.488 µC). In contrast, maca supplements (39A–39C) showed the lowest antioxidant activity in the electrochemical method. Antioxidant activity measured by the FRAP method ranged from 0.256 mmol/g in maca (39A–39C) to 25.010 mmol/g in resveratrol (25B–25E). Antioxidant activity measured by the DPPH method ranged from 0.045 mmol Trolox/g in the Spanish black radish (35A) to 3.501 mmol Trolox/g in resveratrol (25B–25E).

**Figure 4:**
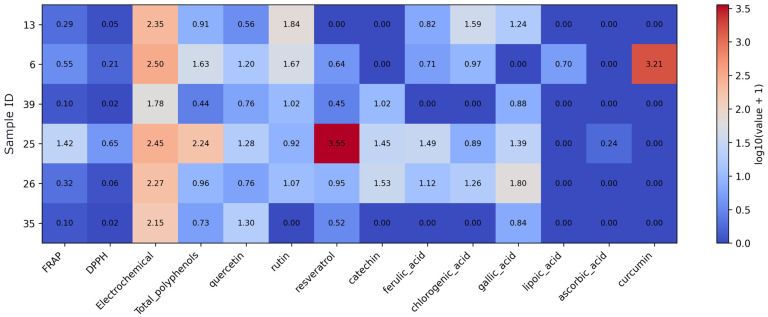
Heatmap of antioxidant assay results and quantified antioxidant compound concentrations in root-based supplements. Note: Values were log10(x + 1)-transformed prior to visualization to reduce dynamic range and preserve zero values. As the data originate from different analytical methods and units, the transformation allows comparative visualization rather than direct quantitative comparison.

Root-derived supplements show relatively low FRAP and DPPH values overall, with consistently higher electrochemical responses across samples (Figure 4). Sample 25 (resveratrol from roots of *Polygonum cuspidatum* and *Fallopia japonica*) stands out with the highest total polyphenols and a very high resveratrol level, together with the strongest FRAP and DPPH responses in this group. Samples 26 (shatavari extract) and 13 (asparagus) are characterized by elevated chlorogenic and gallic acids, whereas rutin is highest in samples 13 (asparagus) and 6 (turmeric). Curcumin is detected only in sample 6 (turmeric), and catechin appears mainly in samples 39 (maca), 26 (shatavari), and 25 (resveratrol from roots of *Polygonum cuspidatum* and *Fallopia japonica*).

Among bark-based supplements (Table 5), the average polyphenol content ranged from 18.123 mg GAE/g in vilcacora (40A–40C) to 39.848 mg GAE/g in pycnogenol (5A–5C). Pycnogenol also exhibited higher antioxidant activity according to three measurement methods. FRAP antioxidant activity was 26.492 mmol/g, DPPH antioxidant activity was 4.195 mmol Trolox/g, while the electrochemical method recorded 150.072 µC.

**Figure 5:**
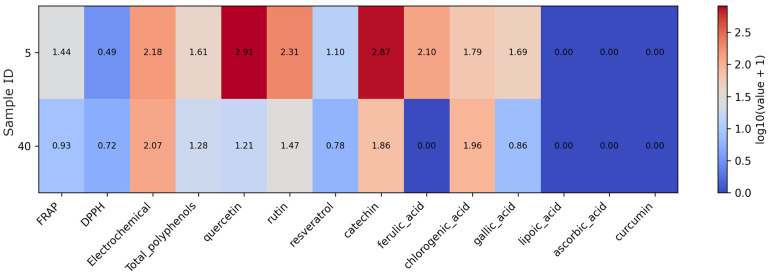
Heatmap of antioxidant assay results and quantified antioxidant compound concentrations for bark supplements. Note: Values were log10(x + 1)-transformed prior to visualization to reduce dynamic range and preserve zero values. As the data originate from different analytical methods and units, the transformation allows comparative visualization rather than direct quantitative comparison.

The two bark products exhibit clearly distinct chemical profiles and assay responses (Figure 5). Supplement 5 containing French maritime bark extract contains substantially higher quercetin, rutin, catechin, and ferulic acid than supplement 40 (containing extract from *Uncaria tomentosa*), which is accompanied by higher FRAP and Electrochemical values. In contrast, DPPH is higher in supplement 40, which also shows higher chlorogenic acid despite lower levels of most other quantified compounds. Lipoic acid, ascorbic acid, and curcumin were not detected in either sample.

Among supplements derived from leaves and herbs (Table 6), the highest average polyphenol content was found in olive (*Olea europaea*) leaf extracts and powders (20A–20C), at 73.991 mg GAE/g. Olive leaf supplements also exhibited the highest antioxidant activity according to three measurement methods, with 7.169 mmol/g (FRAP method), with 1.908 mmol Trolox/g (DPPH method) and 263.48 µC (electrochemical method). The lowest average polyphenol content was observed in broccoli sprout extracts and powders (21A–21C), at 0.988 mg GAE/g. The lutein and zeaxanthin supplement (7A, 7B) displayed the lowest antioxidant activity in three methods, with average values of 0.206 mmol/g (FRAP method), 0.014 mmol Trolox/g (DPPH method) and 129.83 µC (electrochemical method).

**Figure 6:**
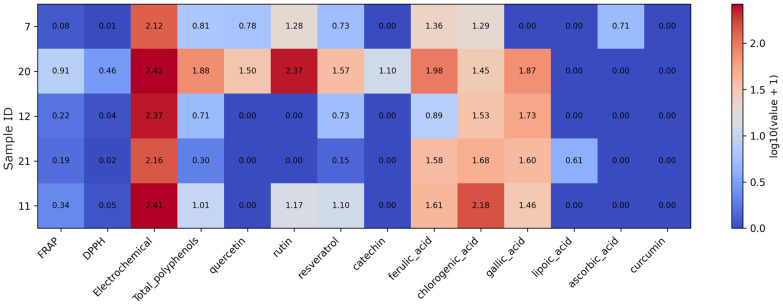
Heatmap of antioxidant assay results and quantified antioxidant compound concentrations in leaf- and herb-based supplements. Note: Values were log10(x + 1)-transformed prior to visualization to reduce dynamic range and preserve zero values. As the data originate from different analytical methods and units, the transformation allows comparative visualization rather than direct quantitative comparison.

The leaf- and herb supplements (Figure 6) show a consistent pattern of high electrochemical responses across all samples, with the strongest values observed for samples 20 (olive leaf) and 11 (watercress). Sample 20 (olive leaf) is characterized by the highest total polyphenols together with elevated rutin, resveratrol, catechin, ferulic acid, and gallic acid. Samples 12 (purslane) and 21 (broccoli) show lower total polyphenols but relatively high chlorogenic and gallic acid levels. Quercetin is detected only in samples 7 (marigold flowers) and 20 (olive leaf), while lipoic acid appears exclusively in sample 21 (broccoli); curcumin is not detected in this group.

Among algae-based supplements (Table 7), spirulina (23A, 23B), derived from the microalga *Arthrospira platensis*, exhibited the highest average polyphenol content (1.319 mg GAE/g) and the highest antioxidant activity measured by the electrochemical method (232.617 µC), and the highest antioxidant activity measured by DPPH method (0.052 mmol Trolox/g). The highest antioxidant activity measured by the FRAP method was observed in chlorella powder (1A–1C), with a value of 0.636 mmol/g. The lowest polyphenol content and antioxidant activity—as measured by both the FRAP and electrochemical methods—were found in supplements derived from *Hematococcus pluvialis* (22A–22E), with values of 1.114 mg GAE/g, 0.144 mmol/g (FRAP), and 51.162 µC (electrochemical method), respectively. The lowest antioxidant activity measured by the DPPH method was found in chlorella (1A–1C) with values of 0.015 mmol Trolox/g.

**Figure 7:**
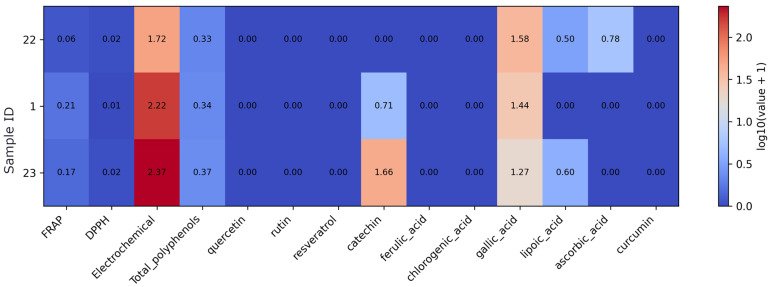
Heatmap of antioxidant assay results and quantified antioxidant compound concentrations in leaf- and herb-based supplements. Note: Values were log10(x + 1)-transformed prior to visualization to reduce dynamic range and preserve zero values. As the data originate from different analytical methods and units, the transformation allows comparative visualization rather than direct quantitative comparison.

Algae-based supplements show low FRAP and DPPH values across all samples, while Electrochemical responses are comparatively higher and differentiate the products (Figure 7). Total polyphenol levels are uniformly low. Gallic acid is the dominant quantified compound in all three samples, whereas catechin is present only in supplements 1 (*Chlorella*) and 23 (spiruline), with the highest level in sample 23 (spiruline). Lipoic acid and ascorbic acid are detected in sample 22 (*H. pluvialis*), and lipoic acid in sample 2 (spiruline). Other quantified compounds are not observed in this group.

Among single-ingredient supplements (Table 8), the average polyphenol content ranged from 0.668 mg GAE/g in alpha-lipoic acid (18A–18C) to 130.845 mg GAE/g in quercetin (30A–30D). Antioxidant activity, as measured by the FRAP method, varied from 0.168 mmol/g in L-glutathione (24A–24C) to 24.504 mmol/g in quercetin (30A–30D). Antioxidant activity, as measured by the DPPH method, varied from 0.038 mmol Trolox/g in alpha-lipoic acid (18A–18C) to 3.689 mmol Trolox/g in quercetin (30A–30D). The lowest antioxidant activity measured by the electrochemical method was found in L-glutathione (24A–24C) at 63.888 µC, while the highest value was observed in quercetin (30A–30D) at 234 µC.

**Figure 8:**
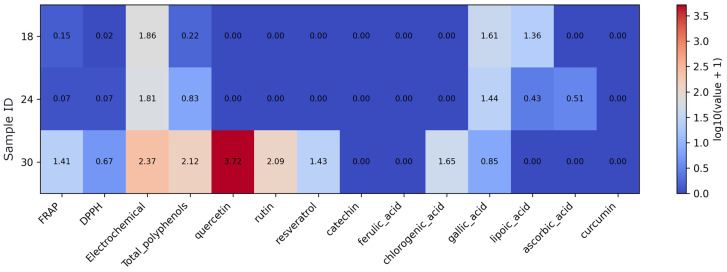
Heatmap of antioxidant assay results and quantified antioxidant compound concentrations in single-ingredient supplements. Note: Values were log10(x + 1)-transformed prior to visualization to reduce dynamic range and preserve zero values. As the data originate from different analytical methods and units, the transformation allows comparative visualization rather than direct quantitative comparison.

In the single-ingredient supplement subset, sample 30 clearly shows the highest antioxidant activity and compound content, with markedly elevated FRAP, DPPH, Electrochemical response, total polyphenols, quercetin, rutin, and resveratrol (Figure 8). Samples 18 (alpha-lipoic acid supplement) and 24 (L-glutathione) display low FRAP and DPPH values and low total polyphenols, with profiles dominated mainly by gallic acid and, in sample 18 (alpha-lipoic acid supplement), also lipoic acid. Other quantified phenolic compounds are not detected in these two products.

Among multicomponent supplements (Table 9), the average polyphenol content ranged from 19.850 mg GAE/g in the red grape skin extract and vitamins supplement (2A) to 126.841 mg GAE/g in the antioxidants supplement (19A–19E). Supplements labeled as number 19 also exhibited the highest antioxidant activity according to the FRAP method, with a value of 19.691 mmol/g, though they had the lowest antioxidant activity based on the electrochemical method (258.19 µC). The highest DPPH antioxidant activity was observed in the red grape skin extract and vitamins supplement (2A) with a value of 0.927 mmol Trolox/g. In contrast the lowest DPPH antioxidant activity was observed in vitamins, minerals and lutein and zeaxanthin (9A, 9B) (0.198 mmol Trolox/g). The lowest FRAP antioxidant activity was observed in the red grape skin extract and vitamins supplement (2A), with a value of 6.981 mmol/g. In contrast, the highest antioxidant activity measured by the electrochemical method was found in the vitamins, minerals, lutein, and zeaxanthin formulation (9A–9B), with a value of 374.395 µC.

**Figure 9:**
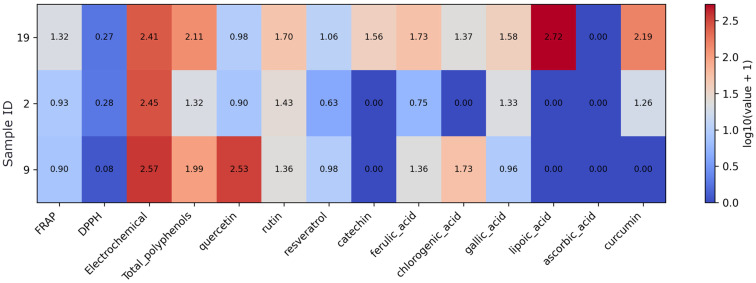
Heatmap of antioxidant assay results and quantified antioxidant compound concentrations in multi-ingredient supplements. Note: Values were log10(x + 1)-transformed prior to visualization to reduce dynamic range and preserve zero values. As the data originate from different analytical methods and units, the transformation allows comparative visualization rather than direct quantitative comparison.

Multicomponent supplements show high electrochemical responses across all samples, with the strongest signal observed for sample 9 (Figure 9). Sample 19 is characterized by the highest total polyphenols together with elevated ferulic, gallic, and lipoic acid and detectable curcumin. Sample 9 shows very high quercetin and chlorogenic acid, whereas sample 2 presents a more moderate and narrower compound profile. Catechin is detected only in sample 19, and ascorbic acid is not detected in this group.

**Figure 10:**
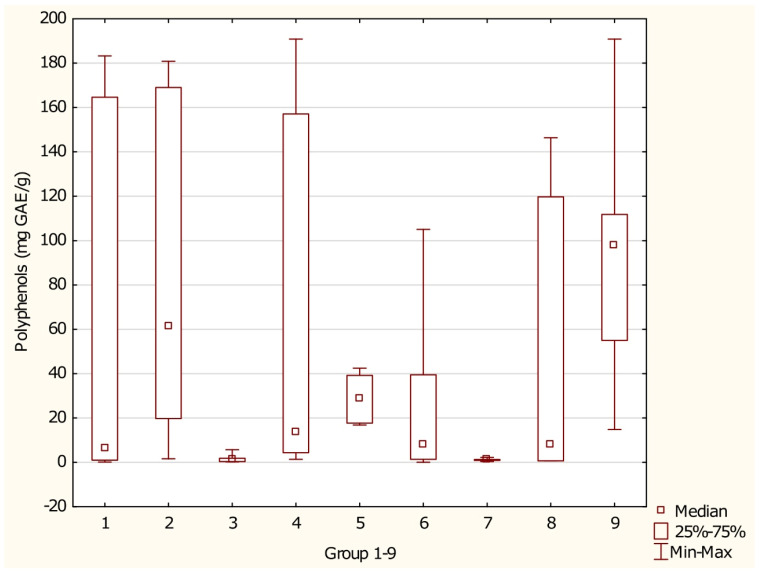
Polyphenol content across distinct groups of antioxidant supplements. Groups of supplements: (1) fruit-based supplements, (2) supplements with coffee or tea, (3) mushroom-based supplements, (4) root-based supplements, (5) bark-based supplements, (6) leaf- and herb-based supplements, (7) algae-based supplements, (8) single-ingredient supplements, and (9) multi-ingredient supplements. *p* 2/7 = 0.0151, *p* 3/9 = 0.0165, *p* 4/7 = 0.0049, *p* 7/9 = 0.0006.

The analyzed supplement groups were evaluated using a non-parametric Kruskal–Wallis rank-sum ANOVA test, followed by post hoc multiple comparisons of mean ranks across all samples. Overall, median polyphenol content ranged from 1.253 mg GAE/g in algae-based supplements (Group 7) to 98.038 mg GAE/g in multi-ingredient supplements (Group 9). Statistically significant differences in polyphenol content were identified between Group 2 (coffee- or tea-based supplements) and Group 7 (algae-based supplements) (*p* = 0.0151), Group 3 (mushroom-based supplements) and Group 9 (multi-ingredient supplements) (*p* = 0.0165), Group 4 (root-based supplements) and Group 7 (*p* = 0.0049), as well as between Group 7 and Group 9 (*p* = 0.0006) (Figure 10). Group 9 exhibited the highest median polyphenol content, while Group 4 showed the widest range. Groups 3 and 7 displayed the narrowest ranges of polyphenol content.

**Figure 11:**
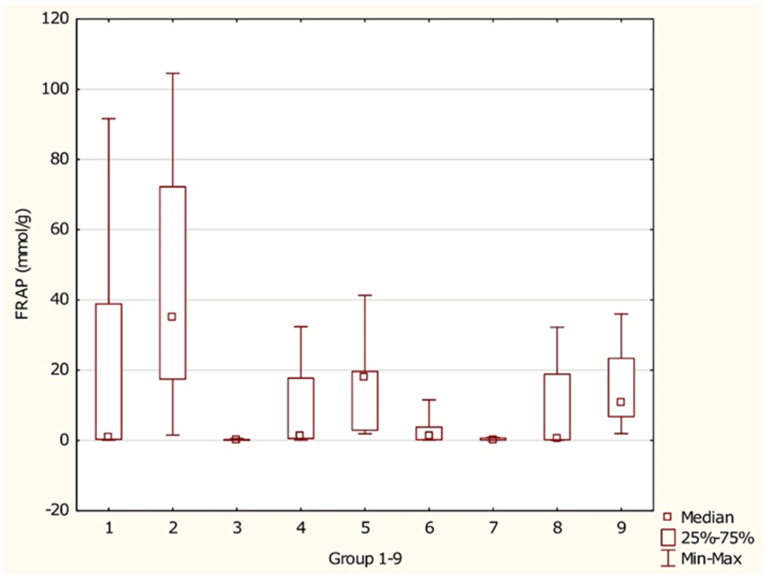
Antioxidant activity (FRAP) in distinct groups of antioxidant supplements. Groups of supplements: (1) fruit-based supplements, (2) supplements with coffee or tea, (3) mushroom-based supplements, (4) root-based supplements, (5) bark-based supplements, (6) leaf- and herb-based supplements, (7) algae-based supplements, (8) single-ingredient supplements, and (9) multi-ingredient supplements. *p* 2/3 = 0.0005, *p* 2/7 = 0.0027, *p* 3/5 = 0.0082, *p* 3/9 = 0.0027, *p* 5/7 = 0.0360, *p* 7/9 = 0.0126.

Figure 11 presents statistically significant differences in antioxidant activity among various supplement groups. The median antioxidant activity ranged from 0.180 mmol/g in mushroom-based supplements to 35.044 mmol/g in coffee- and tea-based supplements. The widest range of antioxidant activity was observed in coffee- and tea-based supplements (Group 2), whereas the narrowest range was recorded in algae-based supplements (Group 7).

**Figure 12:**
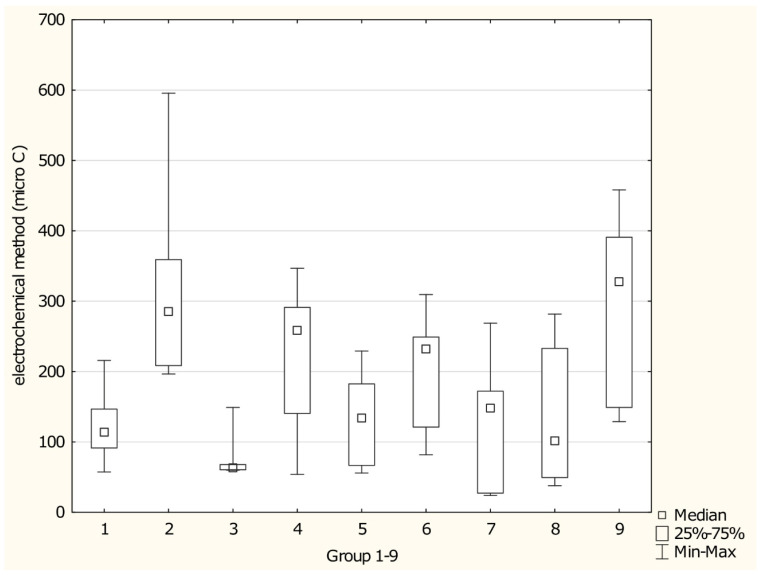
Antioxidant activity measured by the electrochemical method in distinct groups of antioxidant supplements. Groups of supplements: (1) fruit-based supplements, (2) supplements with coffee or tea, (3) mushroom-based supplements, (4) root-based supplements, (5) bark-based supplements, (6) leaf- and herb-based supplements, (7) algae-based supplements, (8) single-ingredient supplements, and (9) multi-ingredient supplements. *p* 1/2 = 0.0421, *p* 2/3 = 0.0059, *p* 3/4 = 0.0177, *p* 3/9 = 0.0073.

Figure 12 illustrates antioxidant activity measured by the electrochemical method across different groups of antioxidant supplements. The median antioxidant activity ranged from 63.073 µC in mushroom-based supplements to 372.470 µC in multi-ingredient supplements. Statistically significant differences in antioxidant activity were identified between Group 1 (fruit-based supplements) and Group 2 (supplements containing coffee or tea) (*p* = 0.042), Group 2 and Group 3 (mushroom-based supplements) (*p* = 0.0059), Group 3 and Group 4 (root-based supplements) (*p* = 0.0173), and Group 3 and Group 9 (multi-ingredient supplements) (*p* = 0.0074). The highest median antioxidant activity was observed in Group 9 (multi-ingredient supplements).

**Figure 13:**
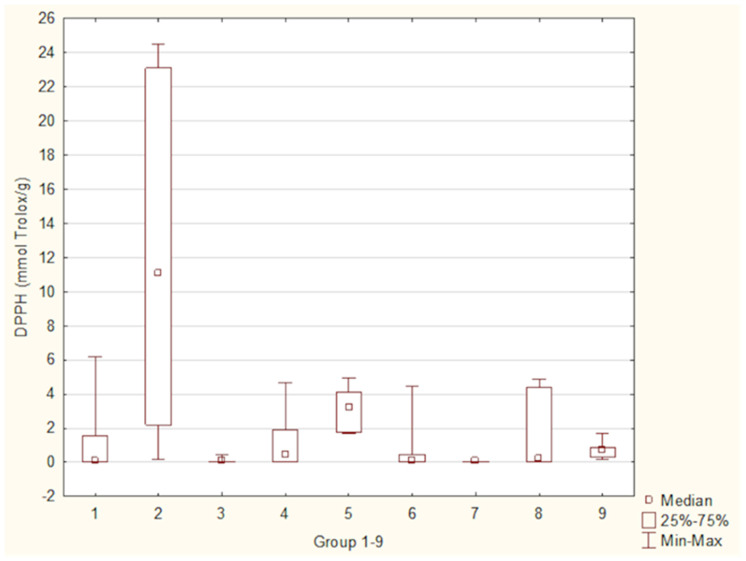
Antioxidant activity measured by the DPPH method in distinct groups of antioxidant supplements. Groups of supplements (1) fruit-based supplements, (2) supplements with coffee or tea, (3) mushroom-based supplements, (4) root-based supplements, (5) bark-based supplements, (6) leaf- and herb-based supplements, (7) algae-based supplements, (8) single-ingredient supplements, and (9) multi-ingredient supplements. *p* 2/7 = 0.0069, *p* 5/7 = 0.0099.

Figure 13 illustrates antioxidant activity measured by DPPH method across different groups of antioxidant supplements. The median antioxidant activity ranged from 0.06 mmol/Trolox/g in algae-based supplements to 11.05 mmol Trolox/g in supplements with coffee or tea. Statistically significant differences in antioxidant activity were identified between Group 2 (supplements with coffee or tea) and Group 7 (algae-based supplements) (*p* = 0.0069). Group 5 (bark-based supplements) and Group 7, *p* = 0.0099.

**Figure 14:**
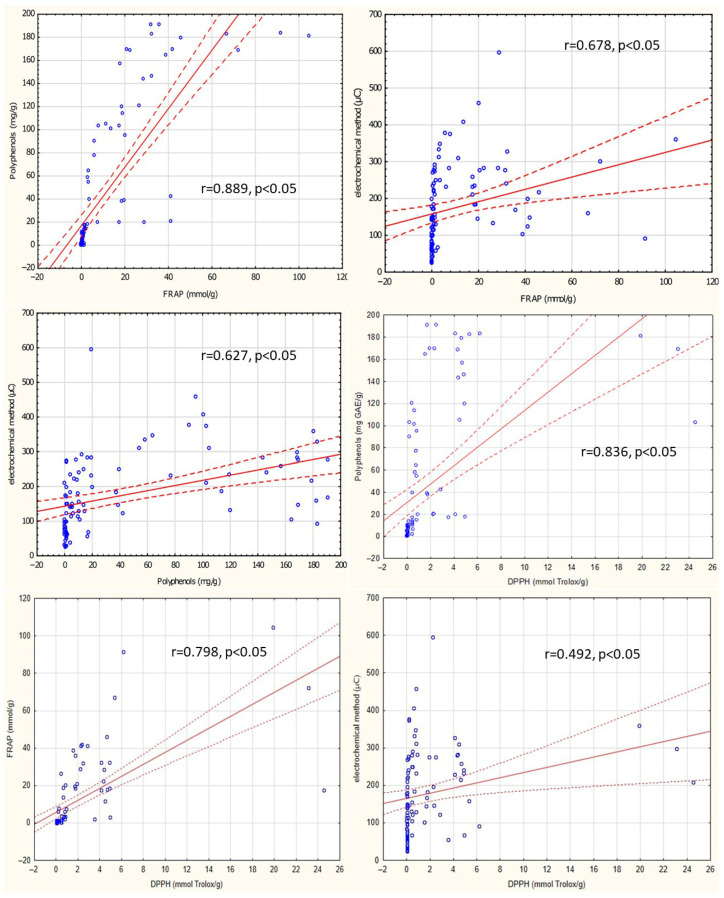
Scatter plot of Spearman’s rank correlation between assayed parameters. Blue dots represent individual data points, solid red line indicates the regression line, dashed lines represent the 95% confidence intervals.

Figure 14 presents scatter plots illustrating Spearman’s rank correlation coefficients between total antioxidant activity, assessed by three methods, the FRAP method, DPPH method and electrochemical methods, and the total polyphenol content of the tested supplements. A strong positive correlation (r = 0.889) was observed between antioxidant activity measured by the FRAP method and total polyphenol content determined by the Folin–Ciocalteu method A moderate positive correlation (r = 0.678) was also observed between the antioxidant activity of the supplements, as measured by the FRAP and electrochemical methods. A moderate positive correlation (r = 0.627) was also observed between total polyphenol content and antioxidant activity measured by the electrochemical method.

A strong positive correlation (r = 0.836) was observed between antioxidant activity measured by the DPPH method and total polyphenol content determined by the Folin–Ciocalteu method. A strong positive correlation (r = 0.798) was observed between antioxidant activity measured by the FRAP method and antioxidant activity measured by the DPPH method. A moderate positive correlation (r = 0.492) was observed between antioxidant activity measured by the DPPH method and antioxidant activity measured by the electrochemical method.

**Figure 15:**
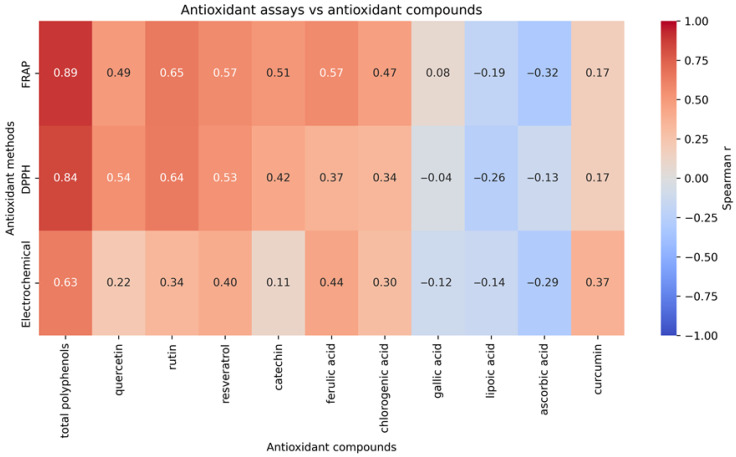
Spearman’s rank correlation heatmap showing relationships between antioxidant assays, total polyphenols, and selected antioxidant compounds calculated across 96 individual dietary supplement samples.

Spearman correlation analysis performed at the individual sample level showed consistent associations between antioxidant assay results and measured compound parameters (Figure 15). The strongest positive correlations were observed between total polyphenols and antioxidant activity measured by FRAP (ρ = 0.89) and DPPH (ρ = 0.84), while the electrochemical method showed a moderate correlation with total polyphenols (ρ = 0.63). Moderate positive correlations were also observed between antioxidant assays and several phenolic compounds, particularly rutin (ρ = 0.64–0.65), resveratrol (ρ = 0.53–0.57), ferulic acid (ρ = 0.37–0.57), and chlorogenic acid (ρ = 0.30–0.47). Catechin showed weaker positive associations (ρ = 0.11–0.51 depending on method). Gallic acid showed near-zero to weak correlations (ρ = −0.12 to 0.08), while lipoic acid and ascorbic acid were negatively correlated with assay responses (ρ from −0.14 to −0.32). Curcumin showed weak positive correlations, most pronounced for the electrochemical method (ρ = 0.37). All reported correlations were statistically significant at the adopted significance threshold.

## 4. Discussion

Dietary supplements constitute a significant segment of both the food and pharmaceutical markets. Unlike pharmaceuticals, they are not subjected to the same rigorous quality control measures, which may lead to variability in their efficacy. In this study, supplements with known or potential antioxidant activity were analyzed for polyphenol content using the Folin–Ciocalteu method and for antioxidant activity using both the FRAP and electrochemical methods. These techniques are widely employed for screening biological materials in various applications.

Both the FRAP and Folin–Ciocalteu methods rely on the single-electron transfer mechanism, in which antioxidant compounds donate an electron to a method-specific reactant, resulting in its reduction and a corresponding color change in the solution. The Folin–Ciocalteu method is used to determine the total phenolic content in test solutions, while the FRAP method quantifies overall antioxidant activity. In this study, a strong correlation between these methods was observed, indicating that polyphenols play a significant role in antioxidant activity as measured by these techniques [21].

In contrast, electrochemical methods allow for direct and rapid screening of antioxidant activity. Their key advantages include high sensitivity, speed, simplicity, and the ability to directly measure electron transfer by antioxidants. Unlike traditional spectrophotometric tests, these methods do not require additional reagents or complex sample preparation, making them more efficient [21,22]. The electrochemical method used in this study enables the measurement of antioxidant activity from compounds with varying redox potentials, allowing for the assessment of both fast-reacting antioxidants, such as ascorbic acid, and slower-reacting antioxidants, such as polyphenols. Notably, the results from the electrochemical method exhibited a strong correlation with both polyphenol content and antioxidant activity measured by the FRAP and moderate positive correlation between electrochemical method and DPPH method. However, while the correlation between FRAP and polyphenols, and between DPPH and polyphenols was strong, the correlation between the electrochemical method and polyphenols was slightly weaker, though still very high. This difference suggests that the nature of molecules contributing to antioxidant activity varies depending on the measurement method. While some overlap exists, polyphenols, as measured by the Folin–Ciocalteu method, represent only one component of the total antioxidant activity detected by the electrochemical method [23].

Fruits are rich in antioxidants, including polyphenols and vitamin C, which contribute to their widespread use in antioxidant supplement production. In this study, grape seed extract supplements exhibited both the highest antioxidant activity and highest polyphenol content. This finding is consistent with the results reported by Krasteva et al. [24], who observed similar polyphenol content in grape seed extracts. Grape seeds contain significant amounts of polyphenols, ranging from 20% to 55% [25]. One of the primary compounds in grape seed extract is proanthocyanidin, known for its anti-inflammatory and antioxidant properties, as well as its role in supporting cardiovascular health [26]. Similarly, apple polyphenol extracts demonstrated high antioxidant activity in this study. Notably, the polyphenol content of apple polyphenol extract in this study was approximately double that reported by Ranjha et al. [27], which may be attributed to differences in extraction methods. The high antioxidant activity of apple extracts is primarily due to their polyphenols, which serve as key secondary metabolites in apple fruit. These include phenolic acids such as gallic acid and p-coumaric acid, flavanols like catechin and epicatechin, dihydrochalcones such as phloretin, and flavonols including quercetin [28].

Among fruit-based supplements, black chokeberry exhibited high antioxidant activity as measured by both the FRAP and electrochemical methods. This high activity is likely due to its substantial phenolic compound content, as chokeberry fruit is particularly rich in anthocyanins, proanthocyanidins, and hydroxycinnamic acids [29].

Açaí berries and goji berries have gained popularity for their health-promoting properties. In particular, goji berries are recognized for their high antioxidant potential, which helps reduce oxidative stress and protect DNA, lipids, and proteins from free radical damage. The antioxidant activity of goji berries is primarily attributed to carotenoids, the fruit’s main pigments, along with phenolic compounds such as caffeic acid, chlorogenic acid, p-coumaric acid, quercetin diglucoside, and rutin [30]. Açaí berries are rich in α-tocopherol, dietary fiber, and polyphenols, including significant amounts of anthocyanins. The antioxidant effects of açaí berries are largely attributed to secondary metabolites such as anthocyanins and proanthocyanidins, particularly cyanidin-3-O-glucoside and cyanidin-3-O-rutinoside [31,32]. Higher polyphenol contents in açaí fruit have been reported in studies by Matta et al. [33] and López de Dicastillo et al. [34], which may be due to variations in growing conditions and extraction methods.

In recent years, apple cider vinegar has gained significant popularity due to its biologically active compounds, with chlorogenic acid being the primary phenolic compound. Studies suggest that apple cider vinegar may help prevent diabetes, hypercholesterolemia, oxidative stress, and even cancer [35]. While encapsulated apple cider vinegar offers greater convenience than its liquid form, the processing involved may affect the quality of the final product. In this study, apple cider vinegar (3A, 3B) exhibited the lowest polyphenol content among fruit-derived supplements. Although previous research has shown that apple cider vinegar can act as an effective antioxidant, demonstrating DPPH free radical scavenging ability [36] and protecting against toxin-induced liver cell damage [37], the polyphenol content and FRAP antioxidant activity in this study did not indicate strong antioxidant potential. This suggests that the production process of this supplement may have reduced both its polyphenol content and antioxidant activity [38].

A slightly higher polyphenol content was observed in lycopene extract from tomatoes, which may be attributed to the higher concentration of natural pigments in tomatoes. Previous studies have highlighted that lycopene from tomatoes has beneficial effects, particularly in the prevention of atherosclerosis, due to its antioxidant properties in lipid-rich environments [39].

The antioxidant activity of plant-derived supplements, especially those based on fruits, can also be influenced by environmental factors such as seasonality and degree of maturity [40]. An analysis of phenolic content at different ripening stages of *Ribes stenocarpum* Maxim fruits revealed that unripe fruits contained significantly higher levels of polyphenols compared to ripe fruits. Consequently, unripe fruits exhibited greater antioxidant activity [41]. Similar observations have been reported in studies on figs, where researchers found a decline in polyphenolic compounds and antioxidant activity as the fruit ripened [42].

Tea and coffee are rich in polyphenolic compounds that provide a range of health benefits, including antioxidant, neuroprotective, and cardioprotective properties. As a result, they are frequently incorporated into antioxidant supplements. The most important polyphenols in tea include epigallocatechin-3-gallate (EGCG), epicatechin-3-gallate (ECG), epigallocatechin (EGC), and epicatechin (EC). In contrast, the main active compounds in coffee consist of caffeine, diterpenes, kahweol, chlorogenic acid, and various phenolic compounds. Chlorogenic acids, caffeine, and melanoidins are considered the primary antioxidants in coffee, with melanoidins being particularly abundant in roasted coffee beans [43].

Among the coffee- and tea-based supplements analyzed, those containing green tea leaf extract exhibited a polyphenol content more than ten times higher than that of green coffee bean supplements. This discrepancy may be attributed to the presence of other compounds responsible for antioxidant activity in coffee, such as melanoidins, which form during the roasting process. Since the coffee supplements in this study were made from green coffee beans, they lacked melanoidins. Additionally, the green tea supplements in this study were standardized for polyphenol content, likely contributing to their higher quality. Similar polyphenol content in green coffee extracts was reported by Mehari et al. [44]. In contrast, the green tea leaf extracts in this study contained approximately 7.5 times more polyphenols compared to those in the study by Tadesse et al. [45]. This difference in polyphenol content may be because green tea does not undergo oxidation, which can alter its polyphenol composition. Variations in extraction methods, plant varieties, and environmental conditions may also explain the differences observed between studies [45].

Mushrooms are commonly included in antioxidant supplements due to their antioxidant properties and polyphenol content, found in both edible and medicinal varieties [46,47]. The key constituents responsible for antioxidant activity in mushrooms include flavonoids, glycosides, polysaccharides, tocopherols, ergothioneine, carotenoids, and ascorbic acid [48]. In this study, the reishi mushroom supplement exhibited the highest polyphenol content, while the chaga mushroom supplement had the lowest. This contrasts with findings from Sharpe et al. [49], where chaga mushroom was reported to have both the highest polyphenol content and the highest FRAP activity, whereas lion’s mane had the lowest. However, in supplements, the bioactive ingredient concentration can vary, which may explain the lower antioxidant activity of chaga mushroom supplements observed in this study. Despite their relatively low polyphenol content and antioxidant activity compared to other supplement groups, mushrooms remain an important source of bioactive compounds, particularly beta-glucans [50], which possess immunomodulatory, anticancer, and antioxidant properties [51].

The root-based supplements analyzed in this study included extracts of *Asparagus officinalis*, *Curcuma longa*, maca, resveratrol, shatavari, and powdered Spanish black radish. Among these, resveratrol-containing supplements exhibited the highest polyphenol content and greatest antioxidant activity. Resveratrol, a polyphenol of the stilbene class, exerts its antioxidant effects both directly, by neutralizing reactive oxygen species, and indirectly, by modulating antioxidant enzymes [52]. In vitro and in vivo studies have emphasized the role of resveratrol in preventing and treating neurodegenerative and cardiovascular diseases [50,52]. High polyphenol content was also observed in *Curcuma longa* root supplements, consistent with findings reported by Quirós-Fallas et al. [53]. Curcumin, the bioactive compound isolated from *Curcuma longa* rhizomes, is well-documented for its antioxidant, antimicrobial, anti-inflammatory, analgesic, and wound-healing properties, as well as its anticancer potential [54]. In contrast, maca supplements derived from *Lepidium meyenii* root powder exhibited the lowest polyphenol content and antioxidant activity within this supplement group. Nevertheless, research highlights maca’s health benefits, including its immunomodulatory, neuroprotective, antiviral, and antioxidant properties [55].

The bark-based supplements analyzed in this study included pycnogenol, an extract from coastal pine (*Pinus pinaster*), as well as extracts and powders of *Uncaria tomentosa* (vilcacora). The antioxidant activity of *U. tomentosa* is attributed to its alkaloid content, along with flavan-3-ols and polyphenols [56]. In contrast, pycnogenol, an extract derived from the bark of French maritime pine (*Pinus pinaster*), consists of a mixture of phenolic compounds, including catechin, epicatechin, taxifolin, procyanidins/proanthocyanidins, flavonoids, and phenolic acids with their glycosides [57,58]. Pycnogenol has been shown to possess four key properties: antioxidant effects, anti-inflammatory effects, positive effects on blood circulation, and enhancement of the extracellular matrix [59]. As a result, pine bark extracts offer a wide range of health benefits, in addition to their potent antioxidant activity [60,61]. These benefits include cardiovascular protection [62], neuroprotective effects [63], as well as anti-inflammatory [57], and anti-diabetic properties [64,65]. Among the bark-based supplements tested, pycnogenol exhibited twice the polyphenol content and significantly higher antioxidant activity, confirming its superior antioxidant properties. This enhanced antioxidant activity can be attributed to the fact that pycnogenol is a patented ingredient, which must meet specific standards, including a required content of catechin and epicatechin, comprising 65–75% of the formulation [60].

Leaves and herbs are natural sources of dietary polyphenols, though some are not suitable for direct consumption and are instead provided as dietary supplements. In this study, olive leaf extracts exhibited the highest antioxidant activity and polyphenol content. The most abundant polyphenols in olive leaves are oleuropein and hydroxytyrosol. Oleuropein is present in high amounts in unprocessed olive fruits and leaves, whereas hydroxytyrosol is more abundant in processed olives and olive oil [66]. The polyphenol content in supplements 7A, 7B, 11A, 11B, and 12A, which contain lutein and zeaxanthin, watercress, and purslane, was approximately ten times lower than that found in *Olea europaea* leaf extracts. Broccoli sprout extracts exhibited the lowest polyphenol content and antioxidant activity. The polyphenol content of the tested supplements was more than ten times lower than that found in broccoli seed and sprout extracts analyzed by López-Cervantes et al. [67]. This discrepancy may be due to differences in the growing conditions of broccoli sprouts, production methods used for the supplements, and variations in composition.

Algae are increasingly utilized in supplement production. The algae-based supplements analyzed in this study included products made from *Chlorella vulgaris*, *Hematococcus pluvialis*, and *Arthrospira platensis*. Algae can serve as an excellent source of antioxidants, though their antioxidant activity can vary significantly depending on cultivation conditions, as demonstrated in previous studies [68]. In this study, algae-based supplements exhibited lower polyphenol content compared to other supplement groups. However, similar polyphenol contents for chlorella and spirulina were reported in a study by Matos et al. [69]. Both spirulina and chlorella possess well-established antioxidant properties. Spirulina activates cellular antioxidant enzymes, inhibits lipid peroxidation and DNA damage, scavenges free radicals, and increases the activity of superoxide dismutase and catalase [70]. Beyond their antioxidant properties, chlorella and spirulina exhibit additional beneficial effects. Research indicates that they have potential applications in the pharmaceutical industry as cholesterol-lowering agents, anticancer agents, immune system boosters, and in the prevention of cardiovascular diseases. Moreover, due to their properties, they can also be used to prevent fat oxidation in food products [71].

Among the single-ingredient supplements tested (alpha-lipoic acid, L-glutathione, and quercetin), quercetin exhibited the highest polyphenol content and antioxidant activity, underscoring its significance as a dietary supplement. Quercetin is a flavonoid known not only for its antioxidant properties but also for its strong antimicrobial, antiviral, and anticancer activities [72]. Clinical studies suggest that quercetin supplementation may be beneficial in the prevention and treatment of chronic diseases, including cardiovascular disorders [73]. Glutathione (GSH) is the most abundant endogenous free radical scavenger in humans. Its primary role is often discussed in the context of mitochondrial protection, where it neutralizes reactive oxygen species (ROS) generated during ATP production, thus preventing oxidative stress damage [73]. However, a randomized, double-blind, placebo-controlled clinical trial investigating the effects of oral glutathione supplementation (500 mg twice daily for 4 weeks) on biomarkers of systemic oxidative stress found no significant changes in oxidative stress markers [74]. This finding suggests that glutathione may have lower bioavailability and antioxidant activity when taken in supplement form. In this study, glutathione exhibited the lowest antioxidant activity in both the FRAP and electrochemical tests, reinforcing its limited role as an effective dietary antioxidant supplement.

Alpha-lipoic acid is a potent free radical scavenger that plays a crucial role in regenerating the reduced forms of other antioxidants, such as vitamins C and E. Although the body naturally produces alpha-lipoic acid, it can also be obtained through dietary sources, including meat, offal, and green vegetables, as well as from supplements [75]. Research indicates that alpha-lipoic acid supplementation is safe and may offer benefits for a wide range of individuals, including smokers, pregnant women, diabetics, and those with cardiovascular diseases [76]. However, in this study, alpha-lipoic acid exhibited the lowest average polyphenol content and antioxidant activity among the tested supplements.

In the multi-ingredient supplement group, formulations exhibited significant variability in ingredient profiles, leading to a wide range of results. The supplement with the highest polyphenol content and antioxidant activity (as measured by the FRAP method) was supplement 19, which contained a comprehensive mix of antioxidant-active ingredients, including plant extracts, fruit extracts, bark and root extracts, as well as vitamins, minerals, and amino acids. This diverse composition likely contributed to positive overall results across all three tests. While a broad range of ingredients can produce a synergistic effect, it is also possible that certain components may interact, potentially diminishing the overall effectiveness of the formulation [77,78]. Supplements 9A and 9B, which contained vitamins, minerals, lutein, and zeaxanthin, exhibited the highest antioxidant activity in the electrochemical method, possibly due to their higher vitamin content relative to polyphenols. Conversely, the supplement containing red grape skin extract, red grape seed extract, vitamin C, polyphenols, and pine bark extract—while rich in polyphenols—demonstrated the lowest polyphenol content and weakest antioxidant activity in the electrochemical method among the multi-ingredient formulations.

This study compared polyphenol content and antioxidant activity across various groups of antioxidant supplements, revealing significant differences between them. Multi-ingredient supplements exhibited the highest median polyphenol content, likely due to the combined effects of diverse ingredients. In contrast, algae-based supplements had the lowest polyphenol content, consistent with previous research indicating that algae typically contain fewer polyphenols [79]. Root-based supplements showed the widest range of polyphenol content, reflecting greater variability, while algae- and mushroom-based supplements displayed the narrowest ranges. In terms of antioxidant activity, coffee- and tea-based supplements exhibited the broadest range, likely due to the variety of antioxidant compounds present in these sources [43]. Overall, multi-ingredient supplements demonstrated the highest levels of both polyphenols and antioxidant activity, suggesting that they may provide superior antioxidant benefits.

The findings of this study demonstrate a substantial degree of variability in the antioxidant capacity of supplements available on the Polish market. This observation is consistent with previous reports indicating discrepancies between declared and actual values in commercially available nutraceuticals [80,81]. Such variability may lead to suboptimal consumer choices and inconsistent effectiveness of supplementation in protecting against oxidative stress. From a regulatory perspective, these findings highlight the need for improved transparency and standardization in the evaluation of antioxidant properties. The introduction of voluntary labeling of antioxidant activity (e.g., FRAP, DPPH) and the standardization of analytical verification methods (e.g., HPLC-based profiling) could support more informed decision-making and enhance the reliability of dietary supplements available on the market.

Heatmap-based profiling revealed pronounced heterogeneity in antioxidant composition and assay responses across supplement categories. Even within the same declared functional class, products differed substantially in both total polyphenol levels and individual compound patterns. This indicates that “antioxidant supplement” labeling does not translate into a uniform chemical or functional profile. A key observation from the heatmaps is that antioxidant assays do not respond uniformly to compound composition. Products rich in specific phenolics (e.g., chlorogenic acid, rutin, catechins, quercetin) tended to show stronger FRAP and DPPH responses, whereas electrochemical signals were often elevated even in samples with relatively low total polyphenols. This supports that different antioxidant assays emphasize different redox mechanisms and compound classes and therefore provide complementary rather than interchangeable information. Although total polyphenol content is often used as a global indicator of antioxidant potential, the heatmap patterns show that assay response is also strongly influenced by compound composition and distribution, not only total concentration.

Distinct compositional signatures were apparent across botanical source groups. Fruit-based and tea-derived supplements were typically associated with catechins, rutin, and chlorogenic acid, whereas coffee-derived products were dominated by chlorogenic acid. Leaf- and herb-based products showed mixed phenolic acid and flavonoid patterns, while mushroom- and algae-based supplements were characterized by narrower profiles frequently dominated by gallic acid and selected non-phenolic antioxidants. Multi-component formulations exhibited the most heterogeneous profiles, reflecting blended raw material sources and formulation strategies.

### 4.1. Strengths

The strength of this study is the large and compositionally diverse set of supplements analyzed, covering multiple raw material categories and formulation types. The study applied a multi-method analytical approach combining FRAP, DPPH, and electrochemical assays with total polyphenol determination and targeted compound profiling. This design enabled cross-method comparison and interpretation of antioxidant responses in relation to compound patterns, providing a robust basis for comparative screening of antioxidant supplements.

### 4.2. Limitations

This study has several limitations. First, the analyzed products represent supplements available on the Polish online market and do not cover the full global range of antioxidant supplements. Therefore, the results should be interpreted as a market-oriented screening rather than a comprehensive survey of all available formulations.

Second, antioxidant activity and polyphenol content were assessed using in vitro assays. These measurements reflect chemical reducing capacity and radical scavenging potential under controlled conditions but do not account for digestion, absorption, metabolism, or bioavailability in vivo. Consequently, the measured antioxidant capacity cannot be directly translated into physiological effects.

Third, some products contained complex multi-component formulations. In such cases, interactions between ingredients may influence overall antioxidant responses, and the applied analytical approach does not allow separation of synergistic or antagonistic effects between components.

Finally, although multiple analytical approaches were applied (Folin–Ciocalteu, FRAP, DPPH, electrochemical assays, and targeted HPLC quantification), the compound panel was limited to selected antioxidants and does not represent the complete phytochemical composition of the products. Other bioactive constituents may also contribute to the measured antioxidant responses.

Moreover, the chromatographic analysis was performed as part of a comparative screening rather than a fully validated quantitative method. Due to the high heterogeneity of supplement matrices, recovery was not determined, as it would require separate validation for multiple representative product types. Therefore, the chromatographic results should be interpreted in a comparative rather than absolute quantitative context.

## 5. Conclusions

This study shows substantial variability in both antioxidant capacity and compound composition among supplements marketed as antioxidant products, with large differences observed in total polyphenols as well as FRAP, DPPH, and electrochemical responses across and within supplement categories. FRAP and DPPH assays demonstrated strong mutual agreement and strong associations with total polyphenols, while relationships with individual compounds were moderate and compound-dependent, being most consistent for rutin, quercetin, resveratrol, ferulic acid, and chlorogenic acid. Electrochemical measurements showed moderate but broader associations with compound profiles, indicating complementary analytical value.

From a practical perspective, these findings indicate that supplements with similar antioxidant claims are not analytically equivalent and should not be compared based on labeling alone. Multi-assay and multi-parameter analytical strategies combining global antioxidant tests with targeted compound profiling provide a more reliable basis for screening, comparison, and quality-oriented evaluation of antioxidant supplements.

## Figures and Tables

**Table 1:** Polyphenol content and antioxidant activity in fruit-based supplements.

No	Sample ID	Supplement Name	*n*	Polyphenols (mg GAE/g)	FRAP Method (mmol/g)	DPPH (mmol Trolox/g)	Electrochemical Method (µC)
				X ± SD Me (Range)	X ± SD Me (Range)	X ± SD Me (Range)	X ± SD Me (Range)
1.	32A, 32B	Açaí berry powder	2	1.285 ± 0.345 1.285 (1.041–1.528)	0.219 ± 0.111 0.285 (0.140–0.297)	0.046 ± 0.003 0.046 (0.044–0.049)	66.8 ± 13.29 66.8 (57.4–76.2)
2.	3A, 3B	Apple cider vinegar powder	2	0.146 ± 0.022 0.146 (0.130–0.162)	0.388 ± 0.133 0.387 (0.293–0.482)	0.014 ± 0.000 0.014 (0.014–0.014)	85.368 ± 14.28 85.368 (75.266–95.47)
3.	15A	Apple polyphenols extract	1	169.695	42.041	2.346	145.566
4.	10A, 10B, 10C	Aronia black chokeberry extract from *Aronia melanocarpa* and powder	3	11.839 ± 2.106 10.329 (9.921–13.756)	1.274 ± 0.228 1.259 (1.053–1.510)	0.249 ± 0.187 0.150 (0.131–0.465)	138.3 ± 21.52 146.65 (113.85–154.4)
5.	34A, 34B	Goji berry powder and extract	2	5.436 ± 1.00 5.436 (4.725–6.146)	0.425 ± 0.09 0.425 (0.357–0.493)	0.273 ± 0.324 0.273 (0.044–0.502)	133.3 ± 15.13 133.3 (122.6–144)
6.	16A, 16B	Grapefruit peel powder	2	7.759 ± 3.502 7.759 (5.282–10.235)	0.640 ± 0.239 0.639 (0.470–0.809)	0.013 ± 0.006 0.013 (0.008–0.018)	120.46 ± 10.309 120.46 (113.17–127.75)
7.	17A, 17B, 17C, 17D	Grape seed extract	4	177.499 ± 8.78 181.06 (164.614–183.257)	60.842 ± 23.74 56.44 (38.862–91.621)	4.419 ± 2.025 4.982 (1.534–6.178)	141.8 ± 57.373 130 (91.35–215.85)
8.	31A, 31B, 31C	Lycopene from tomato extract	3	1.706 ± 2.245 0.433 (0.385–4.298)	0.322 ± 0.421 0.083 (0.074–0.808)	0.018 ± 0.022 0.005 (0.005–0.044)	123.49 ± 51.03 104.78 (84.46–181.25)

X—mean, Me—median, SD-standard deviation, *n*—number of samples.

**Table 2:** Polyphenol content and antioxidant activity in coffee- and tea-based supplements.

No	Sample ID	Supplement Name	*n*	Polyphenols (mg GAE/g)	FRAP Method (mmol/g)	DPPH (mmol Trolox/g)	Electrochemical Method (µC)
				X ± SD Me (Range)	X ± SD Me (Range)	X ± SD Me (Range)	X ± SD Me (Range)
1.	4A, 4B, 4C	Green Coffee Bean including powdered green coffee and extract from coffee	3	13.90 ± 10.65 19.81(1.61–20.29)	23.87 ± 20.27 28.980 (1.53–41.11)	1.547 ± 1.206 2.208 (0.155–2.278)	354.80 ± 211.95 272.30 (196.50–595.60)
2.	36A, 36B, 36C	Green tea extract from leaf	3	150.99 ± 41.91 169.02 (103.09–180.86)	64.734 ± 44.04 72.23 (17.421–104.553)	22.499 ± 2.385 23.093 (19.873–24.531)	288.59 ± 75.75 298.13 (208.523–359.11)

X—mean, Me—median, SD—standard deviation, *n*—number of samples.

**Table 3:** Polyphenol content and antioxidant activity in mushroom-based supplements.

No	Sample ID	Supplement Name	*n*	Polyphenols (mg GAE/g)	FRAP Method (mmol/g)	DPPH (mmol Trolox/g)	Electrochemical Method (µC)
				X ± SD Me (Range)	X ± SD Me (Range)	X ± SD Me (Range)	X ± SD Me (Range)
1.	27A	Chaga mushroom including mycelium *Inonotus obliquus* mycelium powder	1	0.239	0.027	0.051	67.8
2.	37A, 37B, 37C	Lion’s mane mushroom including extract from *Hericium erinaceus* and powdered mushroom	3	1.628 ± 0.215 1.602 (1.427–1.855)	0.183 ± 0.085 0.211 (0.087–0.250)	0.179 ± 0.229 0.047 (0.046–0.444)	64.239 ± 2.088 63.073 (62.993–66.65)
3.	38A, 38B, 38C	Reishi mushroom extract from *Ganoderma lucidum* and powdered mushroom	3	2.534 ± 2.793 1.492 (0.412–5.698)	0.612 ± 0.240 0.18 (0.042–0.510)	0.043 ± 0.007 0.046 (0.035–0.047)	89.796 ± 51.268 60.537 (59.856–148.994)

X—mean, Me—median, SD—standard deviation, *n*—number of samples.

**Table 4:** Polyphenol content and antioxidant activity in root-based supplement extracts and powders.

No	Sample ID	Supplement Name	*n*	Polyphenols (mg GAE/g)	FRAP Method (mmol/g)	DPPH (mmol Trolox/g)	Electrochemical Method (µC)
				X ± SD Me (Range)	X ± SD Me (Range)	X ± SD Me (Range)	X ± SD Me (Range)
1	13A	Asparagus (extract of *Asparagus officinalis*)	1	7.145	0.942	0.111	222.65
2.	6A, 6B, 6C, 6D, 6E	Curcumin (turmeric root extract *Curcuma longa*)	5	41.370 ± 24.48 54.61 (12.730–64.274)	2.518 ± 1.096 3.101 (1.279–3.485)	0.631 ± 0.171 0.671 (0.436–0.823)	312.488 ± 27.53 310.77 (281–346.78)
3.	39A, 39B, 39C	Maca powdered root from *Lepidium meyenii* and extract from root	3	1.730 ± 0.294 1.80 (1.406–2.00)	0.256 ± 0.247 0.132 (0.096–0.541)	0.045 ± 0.003 0.046 (0.041–0.049)	59.775 ± 5.218 62.2 (53.786–63.34)
4.	25B, 25C, 25D, 25E	Resveratrol produced from root and rhizome extract from *Polygonum cuspidatum and Fallopia japonica and* produced by fermentation with *Saccharomyces cerevisiae*	5	173.865 ± 7.004 168.64 (157.073–190.853)	25.010 ± 2.35 20.809 (17.713–32.35)	3.501 ± 1.229 4.130 (1.905–4.673)	283.05 ± 11.41 274.55 (258.66–326.356)
5.	26A, 26B, 26C, 26D	Shatavari extract from root shatavari (*Asparagus racemosus*)	4	8.146 ± 4.22 7.229 (4.332–13.792)	1.074 ± 0.719 0.8515 (0.510–2.084)	0.151 ± 0.218 0.045 (0.035–0.478)	187.25 ± 55.65 179.98 (139.8–249.25)
6.	35A	Spanish black radish powdered root of Spanish black radish (*Raphanus sativus niger*)	1	4.385	0.265	0.045	140.6

X—mean, Me—median, SD—standard deviation, *n*—number of samples.

**Table 5:** Polyphenol content and antioxidant activity in bark-based supplements.

No	Sample ID	Supplement Name	*n*	Polyphenols (mg GAE/g)	FRAP Method (mmol/g)	DPPH (mmol Trolox/g)	Electrochemical Method (µC)
				X ± SD Me (Range)	X ± SD Me (Range)	X ± SD Me (Range)	X ± SD Me (Range)
1.	5A, 5B, 5C	Pycnogenol French maritime bark extract (*Pinus pinaster ait*)	3	39.848 ± 2.412 39.222 (37.809–42.512)	26.492 ± 12.804 19.621 (18.590–41.265)	4.195 ± 0.702 4.111 (3.540–4.936)	150.072 ± 30.085 144.65 (123.066–182.5)
2.	40A, 40B, 40C	Vilcacora extract from bark of *Uncaria tomentosa* and powdered bark	3	18.123 ± 1.50 17.69 (16.880–19.790)	7.486 ± 8.86 2.926 (1.878–17.656)	2.095 ± 0.645 1.762 (1.685–2.838)	117.2 ± 97.14 66.6 (55.8–229.2)

X—mean, Me—median, SD—standard deviation, *n*—number of samples.

**Table 6:** Polyphenol content and antioxidant activity in leaf- and herb-based supplements.

No	Sample ID	Supplement Name	*n*	Polyphenols (mg GAE/g)	FRAP Method (mmol/g)	DPPH (mmol Trolox/g)	Electrochemical Method (µC)
				X ± SD Me (Range)	X ± SD Me (Range)	X ± SD Me (Range)	X ± SD Me (Range)
1.	7A, 7B	Lutein and Zeaxanthin extract from Marigold flowers (*Tagetes* spp.)	2	5.531 ± 6.29 5.531 (9.981–1.081)	0.206 ± 0.140 0.205 (0.106–0.305)	0.014 ± 0.003 0.014 (0.012–0.017)	129.83 ± 67.95 129.83 (81.78–177.88)
2.	20A, 20B, 20C	Olive leaf (extract from leaf of *Olea europaea*, and powdered leaf)	3	73.991 ± 32.92 77.41 (39.489–105.077)	7.169 ± 3.961 6.168 (3.804–11.535)	1.908 ± 2.234 0.784 (0.460–4.481)	263.48 ± 40.742 249.15(231.85–309.46)
3.	12A	Purslane (*Portulaca oleacera* powder)	1	4.115	0.664	0.106	234.6
4.	21A, 21B, 21C	Sulforaphane (broccoli sprout extract, broccoli seed extract and powdered sprout)	3	0.988 ± 0.795 1.416 (0.71–1.478)	0.563 ± 0.594 0.227 (0.212–1.249)	0.039 ± 0.000 0.039 (0.039–0.040)	142.677 ± 58.90 121.1 (97.6–209.33)
5.	11A, 11B	Watercress (dry extract of *Nasturtium officinale* and whole herb powder)	2	9.243 ± 1.39 9.243 (8.258–10.228)	1.203 ± 0.016 1.202 (1.191–1.214)	0.125 ± 0.008 0.125 (0.119–0.131)	258.875 ± 25.42 258.875 (240.9–276.85)

X—mean, Me—median, SD—standard deviation, *n*—number of samples.

**Table 7:** Polyphenol content and antioxidant activity in algae-based supplements.

No	Sample ID	Supplement Name	*n*	Polyphenols (mg GAE/g)	FRAP Method (mmol/g)	DPPH (mmol Trolox/g)	Electochemical Method (µC)
				X ± SD Me (Range)	X ± SD Me(Range)	X ± SD Me (Range)	X ± SD Me(Range)
1.	22A, 22B, 22C, 22D, 22E	Astaxanthin from algae *Hematococcus pluvialis*	5	1.114 ± 0.704 0.930 (0.417–2.242)	0.144 ± 0.054 0.148 (0.062–0.214)	0.049 ± 0.005 0.050 (0.041–0.053)	51.162 ± 54.20 27.3 (24.1–148.05)
2.	1A, 1B, 1C	Chlorella (powder)	3	1.213 ± 0.068 1.25 (1.134–1.253)	0.636 ± 0.22 0.698 (0.392–0.819)	0.015 ± 0.000 0.015 (0.015–0.016)	163.488 ± 13.448 170.266 (148–172.2)
3.	23A, 23B	Spiruline from microalgae *Arthrospira platensis*	2	1.319 ± 0.054 1.319 (1.281–1.358)	0.463 ± 0.305 0.463 (0.247–0.679)	0.052 ± 0.000 0.052(0.052–0.052)	232.617 ± 51.076 232.616 (196.5–268.733)

X—mean, Me—median, SD—standard deviation, *n*—number of samples.

**Table 8:** Polyphenol content and antioxidant activity in single-ingredient supplements.

No	Sample ID	Suplement Name	*n*	Polyphenols (mg GAE/g)	FRAP Method (mmol/g)	DPPH (mmol Trolox/g)	Electrochemical Method (µC)
				X ± SD Me (Range)	X ± SD Me (Range)	X ± SD Me (Range)	X ± SD Me (Range)
1.	18A, 18B, 18C	Alpha-lipoic acid	3	0.668 ± 0.010 0.671 (0.656–0.676)	0.402 ± 0.173 0.494 (0.201–0.510)	0.038 ± 0.000 0.038 (0.038–0.039)	70.75 ± 28.49 71.9 (41.7–98.65)
2.	24A, 24B, 24C	L-glutathione	3	5.732 ± 5.159 4.362 (1.396–11.438)	0.168 ± 0.143 0.13 (0.047–0.326)	0.177 ± 0.224 0.047 (0.047–0.436)	63.888 ± 35.57 24.7 (37.766–104.4)
3.	30A, 30B, 30C, 30D	Quercetin	4	130.845 ± 16.56 131.689 (113.658–146.341)	24.504 ± 6.90 23.647 (18.508–32.213)	3.689 ± 2.055 4.628 (0.625–4.875)	234.76 ± 40.04 236.679 (184.03–281.67)

X—mean, Me—median, SD—standard deviation, *n*—number of samples.

**Table 9:** Polyphenol content and antioxidant activity in multicomponent supplements.

No	Sample ID	Supplement Name	*n*	Polyphenols (mg GAE/g)	FRAP Method (mmol/g)	DPPH (mmol Trolox/g)	Electrochemical Method (µC)
				X ± SD Me (Range)	X ± SD Me (Range)	X ± SD Me (Range)	X ± SD Me (Range)
1.	19A, 19B, 19C, 19D, 19E	Antioxidants made from vitamins, minerals, amino acids, extracts form fruits and herbs, roots and barks; powdered fruits and herbs.	5	126.841 ± 44.02 110.80 (94.93–190.82)	19.691 ± 9.41 23.36 (1.926–35.956)	0.875 ± 0.503 0.807 (0.412–1.725)	258.19 ± 165.44 286.75 (128.85–458.25)
2.	2A	Red grape skin extract and vitamins	1	19.850	7.551	0.927	282.15
3.	9A, 9B	Vitamins, minerals and lutein and zeaxanthin	2	96.675 ± 9.147 96.675 (90.207–103.144)	6.981 ± 1.378 6.981 (6.006–7.955)	0.198 ± 0.013 0.198 (0.189–0.208)	374.395 ± 2.269 374.395 (372.79–376)

X—mean, Me—median, SD—standard deviation, *n*—number of samples.

## Data Availability

The original contributions presented in this study are included in the article. Further inquiries can be directed to the corresponding author.

## Abbreviations

The following abbreviations are used in this manuscript:

DPPH	2,2-diphenyl-1-picrylhydrazyl
EC	Epicatechin
ECG	Epicatechin-3-gallate
EGC	Epigallocatechin
EGCG	Epigallocatechin-3-gallate
FRAP	Ferric Reducing Antioxidant Power
GAE	Gallic Acid Equivalents
GSH	Glutathione
HPLC-DAD	High-Performance Liquid Chromatography-Diode Array Detection
LOD	Limit of Detection
LOQ	Limit of Quantification
PTFE	Polytetrafluoroethylene
ROS	Reactive Oxygen Species
RSD	Relative Standard Deviation
TPTZ	Fe^3+^–2,4,6-tripyridyl-s-triazine

## Appendix A

**Table A1:** Composition of the supplement ingredients.

No.	Sample Symbol	Composition of the Supplement Ingredients	Form	Serving Size/Serving Size Mass	Recommended Daily Dose (Daily Dose Mass)
1.	1A	Powdered *Chlorella vulgaris* including the following: protein 3 g, fiber 0.9 g, chlorophyll 60 mg Iron 6 mg, Zinc 4.5 mg, Viatmin A 135 µg, Vitamin B12 15 µg	powder	2 scoops (8 g)	2 scoops/day (8 g)
2.	1B	Chlorella powder (ruptured cell walls) *Chlorella* sp. Including: protein from Chlorella < 1 g, *Chlorella* sp. (broken cel wall) 1000 mg	capsules	2 capsules (2.04 g)	2 capsules 1–3 times a day (6.132 g)
3.	1C	Chlorella powder (ruptured cell walls) *Chlorella* sp. including: *Chlorella* sp. (broken cell wall) 1000 mg	tablets	1 tablet (0.682 g)	3 tablets/day (2.046 g)
4.	2A	Red grape skin extract (*Vitis vinifera*) 150 mg, Red grape seed extract (*Vitis vinifera*) 72 mg Vitamin C 60 mg, Polyphenols 60 mg, Pine bark extract (3 mg)	tablets	1 tablets (0.619 g)	3 pills/day (1.857 g)
5.	3A	Apple cider vinegar including the following: apple cider vinegar powdered 625 mg	capsule	1 capsule (0.682 g)	1 capsule/day (0.682)
6.	3B	Apple cider vinegar including the following: apple cider vinegar powdered 900 mg	capsule	2 capsules (0.972 g)	2–4 capsules/day (1.944 g)
7.	4A	Green coffee bean including the following: powdered green coffee bean (*Coffea arabica*): 400 mg	capsule	1 capsule (0.552 g)	1–2 capsules/day (1.104 g)
8.	4B	Green coffee bean extract 500 mg including the following: chlorogenic acid 250 mg and caffeine 25 mg	capsule	1 capsule (0.548 g)	1 capsule/day (0.548 g)
9.	4C	Green coffee bean extract 800 mg including the following: chlorogenic acid 400 mg, caffeine 24 mg, chrome 20 µg	capsule	1 capsule (0.799 g)	1–2 capsules/day (1.598)
10.	5A	Pycnogenol-curcumin) including the following: pycnogenol 50 mg	capsule	1 capsule (0.144 g)	1–2 capsule/day (0.288 g)
11.	5B	Pycnogenol-French maritime bark extract (*Pinus pinaser* ait) 30 mg, acerola powdered fruit 100 mg, routine powder (*Sophora japonica* flower) 720 mg	capsule	1 capsule (0.524 g)	1–3 capsule/day (1.572 g)
12.	5C	Pycnogenol-French maritime bark extract (*Pinus pinaser*) 100 mg standardized to oligomeric proanthocyanidins 65 mg	capsules	1 capsule (0.100 g)	1 capsule/day (0.100 g)
13.	6A	Curcumin and piperin includin: Turmeric root extract (*Curcuma longa*) including curcumin 237.50 mg; micronized apple pomace 100 mg; black pepper fruit extract (*Piper nigrum*) 20 mg including piperin 9.50 mg	capsules	1 capsule (0.386 g)	1 capsule/day (0.386 g)
14.	6B	Turmeric root extract (*Curcuma longa*) 475 mg standarized to min 95% curcuminoids (450 mg)	capsules	1 capsule (1.629 g)	2–4 capsules/day(6.516 g)
15.	6C	Liposomal curcumin: curcumin phospholipids 500 mg including 100 mg curcumin, silicon 10 mg	capsules	1 capsle 0.537 g	2 capsules/day (1.074 g)
16.	6D	Turmeric astragalus and gotu kola complex including: Turmeric (*Curcuma longa* (rhizome) 200 mg Astragalus root (*Astragalus membranaceus*) 200 mg Gotu kola (*Centella asiatica*) (aerial parts 200 mg	capsule	1 capsule (0.607 g)	1–2 capsule/day (1.214 g)
17.	6E	Turmeric *Curcuma longa* (rhizome) 720 mg	capsule	1-capsules (0.605)	2-capsules (1.21 g)/day
18.	7A	Lutein and Zeaxanthin: lutein from Marigold Flowers extract (*Tagetes* spp.) 25 mg, Zeaxanthin from Marigold Flowers extract (*Tagetes* spp.) 5 mg	capsule	1 cpsule (0.396 g)	1 capsule/day (0.396 g)
19.	7B	Flower extract from *Tagetes erecta* 100 mg including lutein 20 mg and zeaxanthin 4 mg	capsules	1 capsule (0.184 g)	1 capsules/day (0.184 g)
20.	31A	Tomato extract 66.67 mg including lycopene 10 mg	capsules	1 capsule (0.609 g)	1 capsule/day (0.609 g)
21.	31B	Lycopene tomato extract 66.67 mg including lycopene 10 mg	capsule	1 capsule (0.609 g)	1 capsule/day (0.609 g)
22.	31C	Lycopene including: Lycopene from tomato extract	capsules	1 capsule (0.279 g)	1 capsule/day (0.279 g)
23.	9A	Vitamin A 180 mg, Vitamin C 220 mg, Vitamin E 60 mg, Zinc 7.5 mg, Copper 0.85 mg Selenium 20 µg Lutein 5 mg Zeaxanthin 1 mg	capsules	1 capsule (0.619 g)	2 capsules/day (1.238 g)
24.	9B	Vitamin A 180 mg, Vitamin C 220 mg, Vitamin E 60 mg, Zinc 7.5 mg, Copper 0.85 mg Selenium 20 µg Lutein 5 mg Zeaxanthin 1 mg	capsules	1 capsule (0.619 g)	2 capsules/day (1.238 g)
25.	10A	Aronia black chokeberry fruit (*Aronia melanocarpa*) including powdered fruits 400 mg	capsule	1 capsule (0.573 g)	1–2 capsule/day (1.146 g)
26.	10B	Aronia black chokeberry fruit (*Aronia melanocarpa*) including: black chokeberry fruit extract (20:1) 200 mg micronized black chokeberry pomace 150 mg	capsule	1 capsule (0.295 g)	1 capsule/day (0.295 g)
27.	10C	Aronia black chockebery fruit including: Black chokeberry fruit (*Aronia melanocarpa*) 400 mg	capsule	1 capsule (0.520 g)	1–2 capsule/day (1.040 g)
28.	11A	Dry extract of watercress leaves (*Nasturtium officinale*) DER 20:1–250 mg	capsules	1 capsule (0.294 g)	2 capsules/day (0.588 g)
29.	11B	Watercress-whole herb (*Nasturtium officinale*) 400 mg	capsules	1 capsule (0.478 g)	1 capsule/day (0.478 g)
30.	12A	Purslane (*Portulaca oleacera*) 400 mg	capsule	1 capsule (0.504 g)	1 capsule/day (0.504 g)
31.	13A	Asparagus extract (*Asparagus officinalis*) rhizome and root 170 mg Asparagus stem (*Asparagus officinalis*) 100 mg	capsule	1 capsule (0.315 g)	2 capsules/day (0.63 g)
32.	15A	Apple polyphenols from apple fruit extracts 125 mg	capsules	1 capsule (0.272 g)	1 capsule/day (0.272 g)
33.	16A	Grapefruit peel (*Citrus paradise*) 600 mg	capsules	1 capsule (0.670 g)	1–2 capsules/day (1.34 g)
34.	16B	Grapefruit pectin 1000 mg	capsules	1 capsules (1.535 g)	1–3 capsules/day (4.605 g)
35.	17A	Grape seed extract 100 mg (90 mg polyphenols)	capsules	1 capsule	1–2 capsules/day
36.	17B	Grape seed extract 300 mg including polyphenols 270 mg including oligomeric proanthocyanidins 216 mg	capsules	1 capsule (0.294 g)	1 capsule/day (0.294 g)
37.	17C	Grape seed extract (*Vitis vinifera*) standardized to 90% polyphenols	capsules	1 capsule (0.293 g)	1–2 capsules/day (0.586 g)
38.	17D	Grape seed extract 300 mg including proanthocyanidins 285 mg	powder	300 mg	300 mg/day
39.	18A	Alpha-lipolic acid Including: Alpha-lipolic acid 150 mg	capsules	1 capsule (0.287 g)	1 capsule (0.287 g)
40.	18B	Alpha-lipolic acid Including: Alpha-lipolic acid 100 mg	capsules	1 capsule (0.517 g)	1 capsule (0.517 g)
41.	18C	Alpha-lipolic acid Including: Alpha-lipolic acid 250 mg	capsules	1 capsule (0.500 g)	1 capsule (0.500 g)
42.	19A	Antioxidants including: blackcurrant fiber, rosehip fruit extract (*Rosa canina* L.), acerola fruit extract (*Malpighia glabra* L.; 25% vitamin C, beet root powder (*Beta vulgaris* L.), açaí fruit extract (*Euterpe oleracea*), elderberry fruit extract (*Sambucus nigra* L.), red currant fruit powder (*Ribes rubrum* L.), cherry fruit powder (*Prunus cerasus* L.), cranberry fruit extract (Vaccinium macrocarpon Aiton), goji berry powder (*Lycium barbarum* L.), guava fruit powder (*Psidium guajava* L.), raspberry fruit powder (*Rubus idaeus* L.), baobab fruit powder (*Adansonia digitata* L.), blueberry powder (*Vaccinium corymbosum* L.), strawberry fruit powder (*Fragaria ananassa* Duchesne ex Weston) [30 mg], blueberry fruit powder (*Vaccinium myrtillus* L.) [20 mg], carrot root powder (*Daucus carota* L.) [19.5 mg], Hytolive^®^ olive fruit powder (*Olea europaea* L.) [0.5 mg]	powder	1 scoop (9 g)	1 scoop (9 g)
43.	19B	Antioxidant formula including: Magnesium Ascorbate providing: 150 mg; Vitamin C 141 mg; Magnesium 9 mg; Grape Seed Extract (95% OPC) 30 mg; Beta Carotene (Dunaliella Salina Algae) Equivalent to Vitamin A 833 µg 5 mg; L-Cysteine 50 mg; Zinc (citrate) 5 mg; L-Glutathione 15 mg; Selenium 100 µg; Alpha Lipoic Acid 10 mg; Manganese (citrate) 2 mg; Vitamin B2 (riboflavin) 5 mg; Vitamin B6 (pyridoxine HCl) 5 mg; Copper (citrate) 0.5 mg	capsules	1 capsule (0.595 g)	1 capsule (0.595 g)
44.	19C	Antioxidant including: Vitamin A (beta carotene) 4 mg Vitamin C 240 mg Milk Thistle Seed Extract (80% silymarin) 175 mg; Dandelion Root 150 mg; Choline (choline bitartrate) 60 mg; Inositol 55 mg; N-acetyl-L-cysteine 55 mg. Turmeric rhizome 153 mg; Rosehip fruit 123 mg; Milk thistle seed 87 mg; Tocotrienols 132 mg; Phytosterols 0.2 mg; Lycopene 28 mg; Alpha lipoic acid 2 mg. Cinnamon Bark 1000 mg Prickly Pear Leaf 500 mg Fenugreek Seed 250 mg Astragalus Root 126 mg Burdock Root 62 mg Dandelion Root and Leaf 62 mg. *Bacillus coagulans* 1 billion CFU Inulin 270 mg	capsules and tablets	1 tablet and 6 capsules (4.5 g)	1 tablet and 6 capsules (4.5 g)
45.	19D	Antioxidants including: Turmeric rhizome extract standardized to 95% curcuminoids, Japanese knotweed root extract standardized to 50% trans-resveratrol, Grape seed extract standardized to 95% proanthocyanidins (PAC), Bitter orange fruit extract standardized to 60% hesperidin, Green tea leaf extract standardized to 50% polyphenols, Black elderberry extract standardized to 40% polyphenols and 5% anthocyanins, Rosehip fruit extract standardized to 70% vitamin C, Rhodiola rosea root extract standardized to 1% salidrosides, Ginger root extract standardized to 5% gingerols, Black pepper fruit extract standardized to 95% piperine.	capsules	2 capsules (1.556 g)	2 capsules (1.556 g)
46.	19E	Antioxidant formula including: Vitamin A (as natural beta-carotene (2250 mcg); retinyl palmitate (750 mcg)) 3000 mcg; Vitamin C (as calcium ascorbate) 500 mg; Vitamin E (as d-alpha tocopheryl succinate) 134 mg; Zinc (as zinc glycinate) 10 mg; Selenium (as L-selenomethionine) 50 mcg; Copper (as copper amino acid chelate) 1 mg; Manganese (as manganese glycinate) 4 mg; L-Cysteine HCl 100 mg; Taurine 50 mg; L-Glutathione 25 mg; Pyridoxal 5′-Phosphate 6 mg; Riboflavin 5′-Phosphate 6 mg; Carotenoid Mix (alpha and beta-carotene, lutein, zeaxanthin, cryptoxanthin) 86 mcg; Green Tea Extract (leaf) 31 mg; Red Wine Extract (skin) 15 mg; Pycnogenol^®^ (*Pinus pinaster*) (French Maritime Pine Bark Extract) 5 mg; Ginkgo Biloba Extract 30 mg; Spirulina 20 mg; Gotu Kola Extract (4:1) (aerial part) 10 mg; Milk Thistle Extract (4:1) (seed) 10 mg; Rosehip extract (4:1) (fruit) 20 mg; Rosehip powder (fruit) 6 mg.	capsules	2 capsules (1.552 g)	2 capsules (1.552 g)
47.	20A	Olive leaf extract including: extract from leaf of *Olea europaea* 500 mg	capsules	1 capsule (0.570 g)	1–3 capsules (1.71 g)
48.	20B	Full spectrum olive leaf including powdered olive leaf *Olea europaea*	capsules	1 capsule (0.423 g)	1–2 capsules (0.840 g)
49.	20C	Olive leaf extract including: extract from leaf of *Olea europaea* 420 mg	capsules	1 capsule (0.489 g)	1–3 capsules (1.467 g)
50.	21A	Sulforaphane including: broccoli sprout extract sulforaphane.	capsules	1 capsule (0.474 g)	1 capsule (0.474 g)
51.	21B	Sulforaphane including: Sulforaphane Glucosinolate (from Broccoli seed extract [*Brassica oleracea* L. Italica] in a proprietary blend containing myrosinase Enzyme) 35 mg	capsules	2 capsules (0.848 g)	2 capsules (0.848 g)
52.	21C	Sulforaphane including: powdered broccoli sprouts 100 mg in this sulforaphane 400 mcg	tablets	1 tablet (0.386 g)	1 tablet (0.386 g)
53.	22A	Astaxanthin including: AstaPure Astaxanthin from algae *Hematococcus pluvialis* 4 mg	capsules	1 capsule (0.425 g)	1 capsule (0.425 g)
54.	22B	Astaxanthin including: Astaxanthin from algae *Hematococcus pluvialis* 12 mg	capsules	1 capsule (0.702 g)	1 capsule (0.702 g)
55.	22C	Astaxanthin including: Astaxanthin from algae *Hematococcus pluvialis* 10 mg	capsules	1 capsule (0.790 g)	1 capsule (0.790 g)
56.	22D	Astaxanthin forte including: natural Astaxanthin oil 5% from algae *Hematococcus pluvialis* 80 mg in this 4 mg astaxanthin	capsule	1 capsule (0.607 g)	1 capsule (0.607 g)
57.	22E	Astaxanthin including: natural astaxanthin from algae *Hematococcus pluvialis* 8 mg	capsules	1 capsule (0.378 g)	1 capsule (0.378 g)
58.	23A	Spiruline including: Spirulina from microalgae *Arthrospira platensis*	tablets	1 tablet (0.694 g)	1–2 tablets/day (1.388 g)
59.	23B	Spiruline including: powdered algi *Arthrospira platensis*	tablets	2 tablets (1 g)	4 tablets/day (2 g)
60.	24A	L-glutathione including: L-glutathione reduced 500 mg	capsules	1 capsule (0.542 g)	1 capsule (0.542 g)
61.	24B	L-glutathione including: L-glutathione reduced 100 mg	capsules	1 capsule (0.439 g)	1 capsule (0.439 g)
62.	24C	L-glutathione including: L-glutathione reduced 500 mg	capsules	1 capsule (0.515 g)	1 capsule (0.515 g)
63.	25A	Resveratrol produced by fermentation *Saccharomyces cerevisiae* 250 mg	capsules	1 capsule (0.426 g)	1–2 capsules/day (0.852 g)
64.	25B	Resveratrol including: Vitamin C as ascorbic acid 100 mg, Resveratrol (*Polygonum cuspidatum*) min. 90 mg trans–resveratrol	capsules	1 capsule (0.569 g)	1 capsule/day (0.569 g)
65.	25C	Resveratrol trans including: root and rhizome extract from *Polygonum cuspidatum* 200 mg	capsules	1 capsule (0.197 g)	2 capsule/day (0.394 g)
66.	25D	Resveratrol including: Extract from *Fallopia japonica* 250 mg, standardized for a min. 50% trans-resveratrol (125 mg)	capsules	1 capsule (0.538 g)	1 capsule/day (0.538 g)
67.	25E	Resveratrol including: Japanese knotweed (*Polygonum cuspidatum*) extract 300 mg including trans-resveratrol 150 mg	capsules	1 capsule (0.300 g)	1 capsules/day (0.300 g)
68.	26A	Shatavari including: Extract from shatavari (*Asparagus racemosus*) 10:1, 330 mg	capsules	1 capsules (0.342 g)	1–2 capsules/day (0.684 g)
69.	26B	Shatavari including: Extract from shatavari (*Asparagus racemosus*) 20% (500 mg) including sponins 100 mg	capsules	1 capsule (0.484 g)	1–2 capsules (0.968 g)
70.	26C	Shatavari including: Extract from shatavari (*Asparagus racemosus*) (500 mg) including sponins 150 mg	capsules	1 capsule (0.562 g)	1 capsule/day (0.562 g)
71.	26D	Shatavari including: extract from shatavari (*Asparagus racemosus*) (220 mg) including sponins 88 mg	capsules	1 capsule (0.180 g)	2 capsules/day (0.360 g)
72.	27A	Chaga mushrooms including mycelium *Inonotus obliquus* 400 mg	capsules	1 capsule (0.614 g)	1–2 capsules/day (1.228 g)
73.	30A	Quercetin including dihydrate quercitin 475 mg	capsules	1 capsule (0.698 g)	1 capsule/day (0.698 g)
74.	30B	Quercetin including quercitin 500 mg	capsules	1 capsule (0.688 g)	1 capsule/day (0.688 g)
75.	30C	Quercetin including dihydrate quercitin 75 mg	capsules	1 capsule (0.321 g)	1 capule/day (0.321 g)
76.	30D	liposomal quercetin including: quercetin-phospholipids 250 mg including quercetin 100 mg Zinc 3 mg Selenium 27.5 Silk 12 mg Bromelenin 100 mg Papain 80 mg	capsules	1 capsule (0.533 g)	2 capsules/day (1.066 g)
77.	32A	açaí berry including: açaí Berry fruit extract 100 mg	powder	½ teaspoon (1.5 g)	1 teaspoon/day (3 g)
78.	32 B	Açaí berry including: Açaí berry (*Euterpe oleracea*) 4:1 concentrate 500 mg	capsule	1 capsule (0.660 g)	1–2 capsule/day (1200 g)
79.	34A	Goji berry incuding: Goji berry fruit (*Lycium barbarum*) 1 g	capsule	2 capsule (1.650 g)	2–4 capsule/day (3.3 g)
80.	34B	Goji berry extract including: wolfberry fruit extract (*Lycium barbarum*) 500 mg standardized to 60% polysaccharides	capsules	1 capsule (0.668 g)	1–2 capsule/day (1.336 g)
81.	35A	Spanish black radish including: powdered root of Spanish blsck radish (*Raphanus sativus niger*) 500 mg	capsules	1 capsule (0.654 g)	2 capsules (1.308 g)
82.	36A	Green tea extract including: green tea extract from leaf 500 mg standardized on 60% polyphenols and powdered green tea leaf 500 mg	capsules	1 capsule (0.621 g)	1–2 capsule/day (1.242 g)
83.	36B	Green tea including: green tea leaf extract standardized to contain at least 95% polyphenols and at least 70% catechins and 40% EGC	capsules	1 capsule (0.370 g)	2 capsule (0.74 g)
84.	36C	Matcha including powdered green tea leaves (*Camellia sinensis*)	powder	1 teaspoon (3 g)	1 teaspoon/day (3g)
85.	37A	Lion’s mane including extract from *Hericium erinaceus* 500 mg standardized on polysaccharides 30%	capsules	1 capsule (0.489 g)	1 capsule (0.489 g)
86.	37B	Lion’s mane including extract from *Hericium erinaceus* 500 mg standardized on polysaccharides 50%, 1-3, 1-6 beta-glucans 15% and alpha glucans 5%	capsule	1 capsule (0.516 g)	1–2 capsules (1.032 g)
87.	37C	Lion’s mane including full spectrum powdered mushroom *Hericium erinaceus* 500 mg	capsule	1 capsule (0.775 g)	1 capsule (0.775 g)
88.	38A	Reishi including mushroom extract from *Ganoderma lucidum* (600 mg) standardized on 40% polysaccharides (240 mg)	capsules	1 capsule (0.600 g)	1 capsule (0.600 g)
89.	38B	Reishi including organic mushroom mix from *Ganoderma lucidum*, *Ganoderma applanatum*, *Ganoderma tsugae* (900 mg) including polysaccharides 450 mg, 1-3, 1-6-beta glucans 135 mg and alpha glucans 45 mg	capsules	1 capsule (0.287 g)	3 capsules (0.861 g)
90.	38C	Reishi including extract from *Ganoderma lucidum* 220 mg standardized on 30% polysaccharides and 2% triterpens.	capsules	1 capsule (0.217 g)	2 capsules (0.434 g)
91.	39A	Maca including powdered root from *Lepidium meyenii* 400 mg	capsules	1 capsule (0.584 g)	3 capsules (1.752 g)
92.	39B	Maca including extract from root of *Lepidium meyenii* 500 mg	tablets	1 tablet (0.460 g)	1 tablet (0.460 g)
93.	39C	Maca including extract from root of *Lepidium meyenii* 10:1, 430 mg	capsules	1 capsule (1.01 g)	1 capsule (1.01 g)
94.	40A	Vilcacora including extract from bark of *Uncaria tomentosa* 200 mg including 6 mg oxindole alkaloids	capsules	1 capsule (0.182 g)	1 capsule (0.182 g)
95.	40B	Cat’s claw including powdered bark from *Uncaria tomentosa* 1 g	capsules	2 capsules (1.318 g)	2 capsules (1.318 g)
96.	40C	Full spectrum Cat’s claw including powdered bark from *Uncaria tomentosa* 1 g	capsules	2 capsules (1.32 g)	2 capsules (1.32 g)

**Table A2:** Alphabetical list of dietary supplement manufacturers.

Company Name	Country of Origin
7 Nutrition	Wroclaw, Poland
Aliness	Karczew, Poland
Allnutrition	Opole, Poland
Doctor’s Best (Science-Based Nutrition)	Tustin, CA, USA
Dr Jacobs	Taunusstein, Germany
Fa Super	Rusocin, Poland
Hepatica	Tarnów, Poland
Holand and Barret	London, UK
Jarrow Formulas	Santa Fe Springs, CA, USA
Kenay	Kalisz, Poland
Medverita	Kraków, Poland
My Vita	Legnica, Poland
Nature’s Sunshine	Spanish Fork, UT, USA
Now	Bloomingdale, IL, USA
Ostrovit	Zambrów, Poland
Pharmovit	Płock, Poland
Solgar	Leonia, NJ, USA/UK
Swanson	Fargo, ND, USA
Viridian	Daventry, UK
Yango	Warsaw, Poland

## Appendix B

**Table A3:** Analytical parameters for selected antioxidants assessed with Method 1 (alphabetical order).

Analyte (A–Z)	λ (nm)	RT (min)	R^2^	LOD (µg/mL)	LOQ (µg/mL)
Ascorbic acid	280	3.85	0.999	0.238	0.721
Curcumin	425	42.5	0.999	0.333	1.010
Ferulic acid	320	24.9	0.999	0.314	0.951
Gallic acid	280	8.15	0.999	1.529	4.635
Lipoic acid	330	35.3	0.999	1.322	4.006
Quercetin	360	30.5	0.999	0.133	0.402
Resveratrol	306	28.5	0.999	0.461	1.398
Rutin	360	22.5	0.998	0.438	1.327

Note: LOD and LOQ were calculated using weighted (1/x) regression (area vs. concentration) as 3.3σ/s and 10σ/s.

**Table A4:** Analytical parameters for selected antioxidants assessed with Method 2 (alphabetical order).

Analyte (A–Z)	λ (nm)	RT (min)	R^2^	LOD (µg/mL)	LOQ (µg/mL)
Catechin	280	28.2	0.999	0.892	2.703
Chlorogenic acid	320	29.2	0.999	0.477	1.447

Note: LOD and LOQ were calculated using weighted (1/x) regression (area vs. concentration) as 3.3σ/s and 10σ/s.
